# U.S. dementia care spending by state: 2010–2019

**DOI:** 10.1002/alz.13746

**Published:** 2024-02-27

**Authors:** Amy Lastuka, Michael R. Breshock, Theresa A. McHugh, William T. Sogge, Vivianne Swart, Joseph L. Dieleman

**Affiliations:** ^1^ Institute for Health Metrics and Evaluation University of Washington Seattle Washington USA

**Keywords:** Alzheimer's disease, cost, cost‐of‐illness study, dementia, economics, informal care, United States

## Abstract

**INTRODUCTION:**

Dementia is the fourth largest cause of death for individuals 70 years of age and older in the United States, and it is tremendously costly. Existing estimates of the indirect costs of dementia are dated and do not report on differences across the United States.

**METHODS:**

We used data from multiple surveys to create cost estimates and projections for informal dementia caregiving at the U.S. state level from 2010 through 2050.

**RESULTS:**

In 2019, the annual replacement cost of informal caregiving was $42,422 per prevalent case, and the forgone wage cost was $10,677 per prevalent case. In 2019, it would have cost $230 billion to hire home health aides to provide all this care. If past trends persist, this cost is expected to grow to $404 billion per year in 2050.

**DISCUSSION:**

The cost of informal care varied substantially by state and is expected to grow through at least 2050.

**Highlights:**

- In the United States in 2019, foregone wages due to informal dementia care was $58 billion.
- Replacing informal dementia care with health aides would have cost $230 billion.
- These costs vary dramatically by state, even when assessed per prevalent case.
- These costs are expected to nearly double by 2050.

## 1 BACKGROUND

In addition to being the fourth largest^1^ cause of death for individuals 70 years and older in the United States, dementia is a tremendously costly disease, leading to $80 billion per year in direct medical spending attributable to dementia.^2^ Given the progressive nature of cognitive and functional impairment that accompanies dementia^3^ and the associated caregiving needs, including the cost of informal care substantially elevates these cost estimates. In addition, t number of dementia cases is increasing as the population ages, despite some evidence that dementia incidence is declining.^4^

Unlike many health conditions, the costs of which can be well characterized by medical spending, a large component of the total cost of dementia is attributed to informal care—unpaid care provided by friends or family members—from 30%^5^ to 60%^6^ depending on the methodology used. These costs are not straightforward to measure, with a lack of consensus from published studies on how to value caregiving time.^7^ Three approaches to valuing caregiving time are the opportunity cost method, the replacement cost method (also known as the market price method or proxy good method), and contingent valuation.^8^ The opportunity cost method values the caregiver's alternate use of time and can include both lost wages and lost leisure time; however, leisure time is often excluded due to the lack of a market price for leisure time.^8^ The replacement cost method values the care time at the amount it would cost to replace the informal carer with a paid carer. Contingent valuation is a stated‐preference method in which survey respondents are asked about their willingness to pay to avoid caregiving time or willingness to accept caregiving duty.^9^ Although valuing informal care time is complex, these costs are important to consider, given that the burden of dementia is borne not only by the affected individuals but also by an associated group of caregivers, including many family members.

Several studies have estimated dementia costs at a national level.^5^, ^10^, ^11^ In a pioneering investigation, Hurd et al.^5^ used the Health and Retirement Study^12^ to estimate a total societal cost attributable to dementia in the United States—estimating the total cost in 2010 using two different methods to value the hours of caregiving that were provided. The replacement cost method estimated this cost at $215 billion (95% uncertainty interval [UI] $171–259).^5^ The forgone wage method, which estimates the dollar value of caregivers’ time in terms of lost wages, estimated the cost at $159 billion (95% UI $126–192).^5^ Accounting for population aging but keeping costs per prevalent case constant, Hurd et al. projected that costs would reach $379 billion (95% UI $300–457) (forgone wage approach) to $511 billion (95% UI $408–615) (replacement cost approach) by 2040.^5^

We updated and expanded on the work of Hurd and colleagues by combining caregiving data from the National Health and Aging Trends Study (NHATS),^13^ the Health and Retirement Study (HRS),^14^ and the Behavioral Risk Factor Surveillance System (BRFSS)^15^ to create national estimates of the cost of informal dementia care, as well as estimates for the 50 states and Washington, DC (herein states), and to forecast national informal care costs to 2050. We also disaggregated caregiving time into three components: support for activities of daily living (ADLs), support for instrumental activities of daily living (IADLs), and all other support, which is referred to as supervision. To our knowledge, no study has estimated the cost of informal dementia care at the state level. Our study focuses on informal care, providing a partial estimate of dementia costs from the societal perspective. The ability to compare costs across states is valuable for policymakers, as many policies that affect people living with dementia and their caregivers are set at the state level (e.g., the generosity of home and community‐based services, flexible sick days, family leave coverage), and health care access and quality vary significantly across states.^16^

## 2 METHODS

### 2.1 Estimating the hours of informal care for people living with dementia

We compiled data on caregiving hours from three household surveys: the Behavioral Risk Factor Surveillance System (or BRFSS), which is conducted at the state level, and the Health and Retirement Study (or HRS) and the National Health and Aging Trends Study (or NHATS), which are national surveys. We used five waves of HRS data from 2010 to 2018 and nine waves of NHATS data from 2011 to 2019. The BRFSS caregiver module was administered to various states in 2012, 2013, and 2015 to 2019. Figure S1 shows all states and years for which caregiving data were available. For each data source, we calculated the average weekly caregiving hours per prevalent case of dementia. In the BRFSS survey, the caregiver reports the dementia status of the patient, whereas in HRS and NHATS, cognitive questionnaires are used to classify dementia. These classifications are based on the Diagnostic and Statistical Manual of Mental Disorders, Fifth Edition (DSM‐5) criteria for dementia and include all dementia subtypes. Detailed descriptions of each of these surveys and the methodology used to create caregiving estimates can be found in Section 2.1 of the appendix, and all data sources used in our analysis are listed in Table S1 of the appendix.

**RESEARCH IN CONTEXT**

1. **Systematic review**: The authors reviewed the literature using PubMed. No studies were found that estimated the cost of informal dementia care per prevalent case of dementia at the U.S. state level.
2. **Interpretation**: The cost of informal dementia care to society is large and is increasing as the population ages. Costs vary substantially between U.S. states.
3. **Future directions**: Future studies should investigate the drivers of the informal care cost differences among states.

### 2.2 Estimating the cost of replacing informal caregiving for people living with dementia with a home health aide

The replacement cost method assumes that if the caregiver were not providing free care, the care would be provided by a care worker at market rates. Hurd^5^ and Kelley^4^ use the average cost of a home health aide to value each hour of care, whereas other studies make a distinction between skilled care needed for ADL support versus other tasks.^5^, ^6^

To calculate replacement cost, we allowed reported values for caregiving time of 112 h per week (16 h per day, 7 days per week). Although a survey response of 168 h per week indicates that the care recipient needs round‐the‐clock supervision, we assume that the caregiver would be available overnight and, therefore, the maximum number of hours to be replaced would be 16 per day. After responses were top‐coded to 112 and caregiving estimates were created, outliers were identified using a modified z‐score and removed from analysis. Table S2 lists the outlier states for this model and Figures S2 and S3 report what our cost estimates would be without removing these improbable values.

We then used spatiotemporal Gaussian process regression^1^ (ST‐GPR) to create estimates for caregiving hours per prevalent case for all state‐years from 2010 through 2019. ST‐GPR is a technique that has been adapted for the Global Burden of Disease study^1^ to borrow strength across geography and time when underlying input data are sparse. Its advantages are that it can incorporate all available input data, include viable covariates, and estimate uncertainty as a reflection of the uncertainty of the input data and the estimation method. ST‐GPR is a multi‐step process that first involves estimating a linear regression model. The model specification used was:

(1) $$\log\left(hours_{SY}\right)=\;\alpha +\beta_{I}I_{HRS}+\;\beta_{2}Educ_{SY}+\beta_{3}Diabetes_{SY}$$

where $hours_{SY}$ is the average caregiving hours per prevalent case of dementia for state S and year Y, $I_{HRS}$ is an indicator for estimates based on the HRS survey, $Educ_{SY}$ is the mean years of education for state S and year Y, and $Diabetes_{SY}$ is the age‐standardized diabetes mellitus prevalence for state S and year Y. Smoking prevalence and the prevalence of high blood pressure were also considered for this model but were not found to be significant predictors of caregiving time. The linear regression model results can be found in Table S3. The results of the linear model were then smoothed over space and time to create final estimates.

The cost per hour of care was derived from home health aide wage data and data from the Genworth Cost of Care Survey.^17^ The cost of a home health aide varies significantly across the United States; in the 2018 Genworth Cost of Care Survey, the hourly cost ranged from $16 in Louisiana to $30 in Hawaii. More detail on home health aide costs can be found in Section 2.5 of the appendix. The total replacement cost estimates for people living with dementia were produced by multiplying the annual caregiving hours by the hourly cost for each state‐year. All costs are adjusted for inflation by converting to 2019 U.S. dollars using the gross domestic product (GDP) implicit price deflator from the Bureau of Economic Analysis.^18^

### 2.3 Estimating wages forgone because of informal caregiving for people living with dementia

We followed the approach of Hurd et al.^5^ to estimate forgone wages due to informal caregiving. With this approach, each hour of caregiving time is valued at the rate of the expected wage of the caregiver, up to the first 40 h per week of care. Additional hours of care are not assigned a monetary value; therefore, lost leisure time is not captured in this analysis. Although lost leisure time is an important aspect of caregiving, it is more difficult to value and was, therefore, not included in this work.

Top‐coding the caregiving hours to 40 h per week affects the point estimates created for average caregiving hours per prevalent case of dementia. Therefore, outlier analysis was conducted a second time, with results reported in Table S4 of the appendix. We fit a linear model of caregiving hours using Equation 1 and then used ST‐GPR to create estimates for caregiving hours per prevalent case for all state‐years from 2010 through 2019. The linear regression model results can be found in Table S5. Caregiving hours were then multiplied by the expected wage for each caregiver. The expected wage is the average wage for people with the caregiver's demographic characteristics (age, sex, and education level) in a given state and year, scaled by the labor force participation for that demographic group to account for the fact that some of the caregivers would not have been in the workforce.

Average wages by state, year, and demographic group (age, sex, and education level) were calculated from the Current Population Survey.^19^ Each caregiver was assigned an expected wage based on their age, sex, and education level. More detail on the expected wage methodology can be found in Section 2.6 of the appendix. For each state‐year, an average hourly cost was estimated as the hours‐weighted average wage. Outlier analysis was conducted for these expected wage estimates, with results reported in Table S6 of the appendix. ST‐GPR was used to fill in estimates for missing state‐years. The linear model specification was:

(2) $$\log\left(wage_{SY}\right)=\;\alpha +\beta_{I}income_{SY}$$

where $wage_{SY}$ is the hours‐weighted average wage for caregivers in state S and year Y, and $income_{SY}$ is the mean household income for state S and year Y. The model results can be found Table S7.

Our modeling approach for both replacement cost and forgone wage cost produced cost estimates per prevalent case of dementia. To estimate total costs at the state or national level, we multiplied these per‐case costs by prevalence estimates from the Global Burden of Disease study.^20^ These prevalence estimates include the following dementia subtypes: Alzheimer's disease, vascular, Lewy body, brain injury, alcohol‐related, frontotemporal, and mixed; however, they are not categorized explicitly by subtype due to concerns that the underlying pathology is not well matched to clinical diagnoses.^21^

### 2.4 Costs attributable to dementia

Not all caregiving hours provided to a person with dementia are attributable to dementia. To estimate the percentage of caregiving hours that are attributable to non‐dementia comorbidities, we followed the methodology described by Dieleman and colleagues^2^ for attributable costs. First, we estimated a log‐linear relationship between caregiving hours and comorbidities as follows:

(3) $$\log\left(Hours_{i}\right)\;=\;\alpha +\sum_{j}\beta_{j}I\left(X_{j}\right)+\varepsilon$$

where $Hours_{i}$ are the weekly caregiving hours provided to person i, and X is an indicator variable for each comorbidity j.

We then accounted for the fraction of caregiving hours that are attributable to comorbidity j($AF_{j})$ using the following equation:

(4) $$A\;F_{j}=p_{j}\;\left(e^{\beta_{j}}\;-1\right)$$

where $\beta_{j}\;$ is the linear model coefficient for health conditions j from Equation 3, and $P_{j}$ is the conditional probability of dementia and health condition j co‐occurring. Adding the attributable fractions from each comorbidity and subtracting this total from 100% resulted in the caregiving fraction attributable to dementia. Table S8 shows the attributable fraction for each comorbidity modeled.

A national attributable fraction was estimated using NHATS data, and that attributable fraction was used across all states. BRFSS data do not have information about the care recipient's comorbidities, so no state‐level variation of the attributable fraction was estimated.

### 2.5 Estimating care hours by activity

One difficulty in measuring caregiving time is defining the tasks that constitute caregiving. Surveys that measure caregiving face a trade‐off between asking narrowly defined questions that lead to more precise estimates versus asking broader questions that are more inclusive of all caregiving time but lead to less precise estimates. A commonly used taxonomy of caregiving tasks categorizes all tasks as ADL support, IADL support, or supervision. The Resource Utilization in Dementia (RUD) instrument,^22^ which is used in many dementia caregiving studies, asks caregivers about each of the three categories: ADL support, IADL support, and supervision.

Our estimates include caregiving time for all activities. In contrast, the estimates from Hurd and colleagues^5^ are informed by the Health and Retirement Study, which only asks about ADL and IADL support. Therefore, to enable comparisons of our results and estimates based on the HRS, we used data from the GERAS‐US study,^23^ which fielded the RUD instrument, to estimate the proportion of time spent on each category.

### 2.6 Cost decomposition

To explore the wide variation in informal care costs, we decomposed the per capita annual cost into four terms as shown in Equation 5.

(5) $$Cost\;=\;\frac{Prevalence}{Prevalence_{AS}}*Prevalence_{AS}*Hourly\;cost*Caregiving\;hours$$

The first term, which we refer to as age profile, is a ratio of the raw dementia prevalence to the age‐standardized dementia prevalence. The remaining terms are age‐standardized dementia prevalence, hourly cost, and caregiving hour components using a Das Gupta decomposition.^24^

### 2.7 Cost projections

We produced two forecasts for dementia costs. First, in the low‐growth scenario, we held costs per prevalent case constant at the average cost from 2010 through 2019. We then used dementia prevalence projections created for the Global Burden of Disease project to forecast costs to 2050.^20^ The dementia prevalence forecasts account for population growth and aging, as well as forecasted trends in several modifiable risk factors. We refer to this as the low‐growth scenario because the only growth in cost arises from the increase in dementia prevalence.

Second, in the high‐growth scenario, we estimated a linear growth rate for costs per prevalent case based on the cost estimates from 2010 through 2019. In the high‐growth scenario, both costs per prevalent case and dementia prevalence are increasing.

## 3 RESULTS

### 3.1 Hours and wages

We found that the cost of a home health aide increased slightly when accounting for inflation. In 2019 U.S. dollars, the national average hourly cost was $23.02 (95% uncertainty interval [UI] 22.63–23.42) in 2010 and rose to $24.69 (95% UI 24.37–25.01) in 2019. The expected wage of a caregiver also saw a small increase, rising from $12.27 (95% UI 12.03–12.51) in 2010 to $12.77 (95% UI 12.51–13.02) in 2019. The number of caregiving hours per prevalent case that were attributable to dementia rose modestly over time. When allowing up to 112 h per week of care, caregiving hours increased from 31.4 (95% UI 26.3–36.5) in 2010 to 33.0 (95% UI 27.6–38.4) per week in 2019. When capping caregiving time at 40 h per week, caregiving hours were relatively stable, ranging from 16.4 (95% UI 13.3–19.5) in 2010 to 16.1 (95% UI 13.1–19.1) per week in 2019.

Home health aide cost, expected caregiver wages, and caregiving hours attributable to dementia varied widely by U.S. state. In 2019, the cost of a home health aide varied from $19.45 (95% UI 18.41–20.48) in Louisiana to $32.40 (95% UI 30.38–34.44) in North Dakota. Caregivers’ expected hourly wage varied from $10.98 (95% UI 10.76–11.19) in Mississippi to $14.48 (95% UI 14.18–14.76) in Washington, DC. Weekly caregiving hours per case ranged from 19.3 (95% UI 16.1–22.4) in Washington, DC, to 48.7 (95% UI 40.6–56.5) in West Virginia for the replacement cost model, and from 7.3 (95% UI 6.0–8.8) in Washington, DC, to 25.4 (95% UI 20.7–30.1) in West Virginia for the forgone wage model.

### 3.2 Costs per prevalent case

**FIGURE 1:**
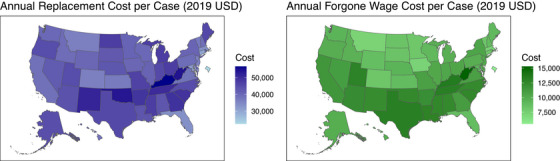
Map of replacement costs and forgone wage costs per prevalent case of dementia.

**FIGURE 2:**
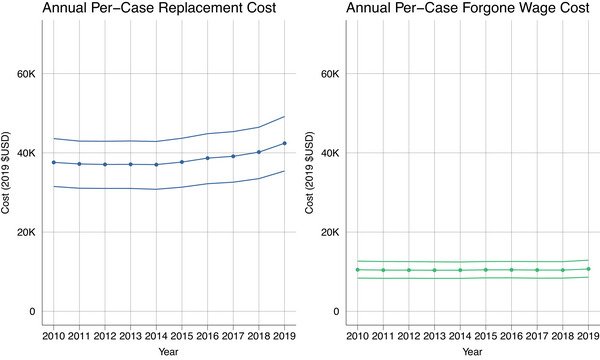
Costs per prevalent case from 2010 through 2019. All costs are in 2019 U.S. dollars.

We found that 72% (95% UI 62–82) of caregiving hours were attributable to dementia. Unless otherwise noted, all estimates herein have been adjusted to reflect costs attributable to dementia. The national estimates for indirect cost per patient in 2019 were $42,422 (95% UI 35,422–49,181) using replacement cost and $10,677 (95% UI 861112,904) for forgone wages. The estimated cost of caregiving varied substantially by state (Figure 1). The replacement cost per case ranged from $22,436 (95% UI 18,705–26,214) in Washington, DC to $56,684 (95% UI 47,043–66,416) in Kentucky. The forgone wage cost per case varied from $5,534 (95% UI 4451–6735) in Washington, DC to $15,297 (95% UI 12,372–18,417) in West Virginia. –Figure 2 shows the per case cost estimates from 2010 to 2019 for both the replacement cost and forgone wage approaches. Table S9 shows per‐case cost estimates and confidence intervals for all states.

### 3.3 Adjusting for care activities

Using the caregiving activity proportions derived from GERAS‐US, we found that 25% of time was spent on ADL support, 42% on IADL support, and the remainder on supervision. Table 1 shows our main caregiving estimates, which reflect all caregiving activities, along with two subsets of caregiving tasks: ADL support only and ADL and IADL support. The replacement cost per prevalent case ranged from $10,435 (95% UI 8713–12,097) for ADL support only to $38,557 (95% UI 35,371–41,802) for all caregiving activities. This wide range highlights the sensitivity of caregiving cost estimates to how caregiving is defined.

**TABLE 1:** Costs per prevalent case in 2019 by activity type.

	ADL support	ADL + IADL support	All caregiving
**Replacement cost**	$10,435 (8713–12,097)	$28,117 (23,477–32,596)	$42,422 (35,422–49,181)
**Forgone wage cost**	$2,626 (2118–3174)	$7,077 (5707–8553)	$10,677 (8611–12,904)

### 3.4 Cost decomposition

**FIGURE 3:**
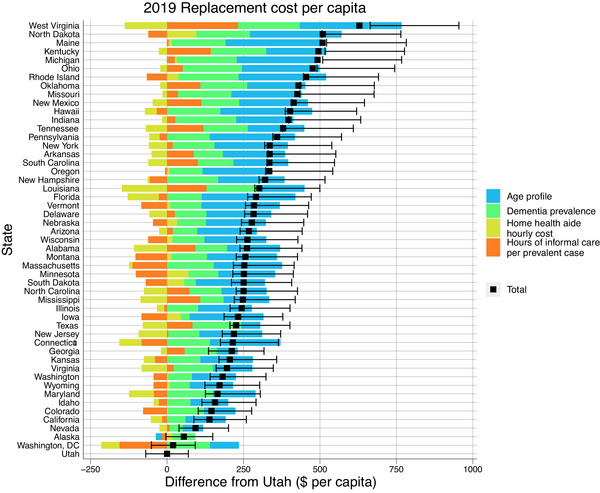
Das Gupta decomposition of per‐capita replacement costs. Costs are relative to the per‐capita cost in Utah.

Figure 3 shows the variation in per capita replacement costs by comparing each state's cost to that in Utah, the state with the lowest per capita cost; it also identifies the contributions of age profile, dementia prevalence, home health aide hourly cost, and hours of informal care per prevalent case to the cost difference. Across states, age profile was found to be the largest contributor to per capita cost differences. Although Washington, DC, had the lowest replacement cost per prevalent case, Utah's per capita costs were lower due to a younger age profile and lower age‐standardized dementia prevalence rate. Despite having a relatively old population, Florida was not in the top 10 most expensive states per capita, largely due to its lower hourly cost of a home health aide. A cost decomposition for per capita forgone wage costs is shown Figure S4.

### 3.5 Total costs

**FIGURE 4:**
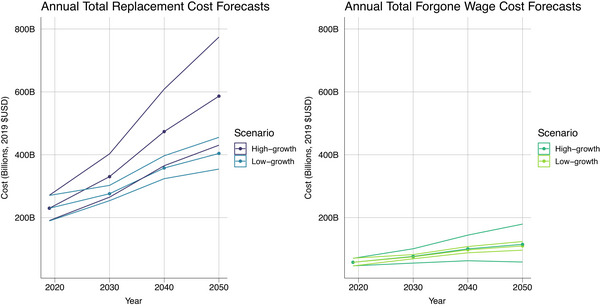
Total cost forecasting through 2050. All costs are in 2019 U.S. dollars.

Figure 4 shows the estimated cost of informal dementia care for 2019, as well as the projected annual costs in both the low‐growth and high‐growth scenarios. In 2019, the total annual replacement cost of informal dementia care was $230 billion (95% UI 189–271), and the total annual cost in terms of caregivers’ forgone wages was $58 billion (95% UI 46–71).

In the low‐growth scenario, in which costs per prevalent case remain constant, the total replacement cost of informal dementia care is projected to reach $404 billion (95% UI 354–455) by 2050, while the total forgone wage cost is projected at $110 billion (95% UI 96–124). Under the high‐growth scenario, in which costs per prevalent case are increasing over time, the total replacement cost of informal dementia care is expected to be $586 billion (95% UI 430–774) by 2050, and the total forgone wage cost is estimated to be $115 billion (95% UI 59–179).

**FIGURE 5:**
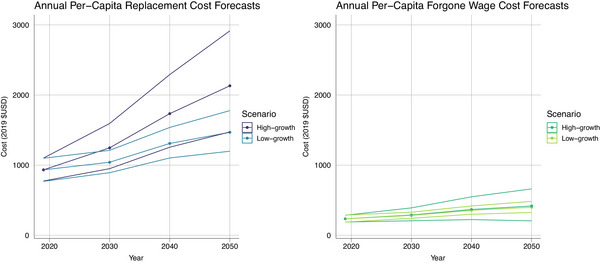
Per capita cost forecasting through 2050. All costs are in 2019 U.S. dollars.

Figure 5 shows the projected per capita annual costs of informal dementia care through 2050. In the low‐growth scenario, the per capita cost in terms of forgone wages is expected to increase from $235 (95% UI 188–288) to $399 (95% UI 326–483) by 2050, whereas in the high‐growth scenario the per capita cost is forecasted to nearly double, reaching $418 (95% UI 205–662) by 2050. The replacement cost is projected to increase from $933 (95% UI 769–1099) to $1468 (95% UI 1199–1778) in the low‐growth scenario and could reach $2131 (95% UI 1473–2,915) in the high‐growth scenario. The projected values can also be found in Tables S10 and S11.

## 4 DISCUSSION

This research updates national cost estimates for informal dementia care in the United States, and it provides the first cost estimates by U.S. state. Our estimates indicate that the care provided by family and friends in 2019 would have cost almost a quarter of a trillion dollars if it had been provided by home health aides. The estimated cost in terms of forgone wages of these caregivers was smaller but still over $50 billion. A recent study^2^ reported that direct spending attributable to dementia in the United States was $79.2 billion in 2016, and our 2016 estimates were $195 billion (95% UI 162–230) for the replacement cost and $53 billion (95% UI 43–64) for the forgone wage cost. Combining these medical spending estimates indicates that, with a replacement cost framework, the indirect costs of dementia account for 71% (95% UI 67–74) of the total cost; with a forgone wage framework, the indirect costs make up 40% (95% UI 35–45) of the total cost.

These estimates of the relative contribution of informal care to total dementia costs fall within the ranges found in other studies. Wimo and colleagues found that informal care makes up 44% of total costs in high‐income countries and rises to 85% of the total cost for low‐income countries.^25^ Even within Europe there is significant variation in the level of reliance on informal care, with informal care making up ≈75% of total cost in southern Europe and only 25% of total cost in the Nordic region.^26^ These differences are likely due to a mix of cultural factors and variation in the amount and types of support policies in place for caregivers.^27^

Our forecasts illustrate the impact of rising dementia prevalence, with total costs increasing by over 50% between 2019 and 2050, even if costs per prevalent case stay constant. Our state‐level estimates showed that costs per prevalent case varied considerably by state. The largest driver of cost differences between states was the age profile of the state, with the number of caregiving hours per prevalent case also accounting for a substantial portion of cost differences.

Our estimate of the cost per prevalent case for 2019 was $42,422 (95% UI 35,422–49,181) for replacement cost. Our replacement cost estimate was slightly higher than the estimate from Hurd and colleagues of $32,456 (95% UI 24,658–40,255) when adjusted to 2019 U.S. dollars. A key difference was that we accounted for all categories of care, whereas the estimates from Hurd and colleagues accounted for only ADL and IADL support. When we scaled our replacement cost estimate to reflect only ADL and IADL support, it was reduced to $28,117 (95% UI 23,477–32,596), which was slightly below the estimates from Hurd and colleagues. The forgone wage estimate from Hurd and colleagues was $15,403 (95% UI 11,254–19,552) when adjusted to 2019 U.S. dollars.^5^ This is significantly larger than our forgone wage estimates of $10,677 (95% UI 8611–12,904) per prevalent case, due to our decision to cap forgone wage hours at 40 per week. Results from a forgone wage model that allowed up to 112 h per week are shown in Tables S12 and S13 and were similar to the estimates from Hurd and colleagues. These findings indicate that informal caregiving needs for people living with dementia have not changed substantially since the estimates from Hurd and colleagues were produced and highlight the contribution of supervisory time to the overall cost of informal dementia care.

We disaggregated national estimates for informal dementia care to the state level by using data from BRFSS, NHATS, and HRS. The most expensive state (Kentucky) had a replacement cost that was 2.5 times higher than the least expensive state (Washington, D.C.). Using a forgone wage approach led to similar variation in cost by state, with West Virginia (the most expensive state) being 2.8 times more expensive than Washington, DC (the least expensive state). These differences were driven largely by care hours. With our replacement cost model, which allowed for up to 16  per day of care, the estimated attributable hours of care per case in 2019 were 19.3 (95% UI 16.1–22.4) for Washington, DC and 42.6 (95% UI 35.5–49.4) for Kentucky. Per‐case indirect costs are relatively low in high‐income states such as Washington, DC, Connecticut, and Maryland, which may reflect a heavier reliance on formal (paid) care in these states.

In addition to per‐case cost estimates, per capita costs provide insight into the total indirect cost burden for each state. We decomposed per capita replacement cost into four components: the state age profile, the dementia prevalence in that state, the hours of care needed, and the cost per hour to examine the contribution of each component to the overall cost. In per capita terms, Utah had the lowest replacement cost. This was driven by both its young age profile and its low age‐standardized dementia prevalence rate. Only Alaska had a younger population than Utah, and no state had a lower age‐standardized dementia prevalence rate. West Virginia had the highest per capita cost of informal care, despite having lower than average wages.

We created two dementia cost forecasts based on two different scenarios: in the first scenario, the costs per prevalent case were assumed to be stable, whereas in the second scenario, costs per prevalent case were assumed to increase linearly over time, following the trend over the past 10 years. The large difference between these projections highlights the impact that per‐case cost growth could have on total costs. In addition to uncertainty around costs per prevalent case, it is important to note that new disease‐modifying treatments for Alzheimer's disease have the potential to change the trajectory of informal care needs for that subpopulation significantly.^28^ Although slowing disease progression will provide an increase in quality‐adjusted life years,^29^ increased life expectancy and decreased transition to skilled nursing facilities may lead to higher demand for informal care. ^30^ Another caveat to apply to these forecasts is that shrinking family sizes in the United States could lead to a shortage of informal caregivers. Policymakers may want to consider scenarios in which the supply of caregivers does not meet the forecasted demand for informal care.

Our analysis has several limitations. First, assigning dementia status to individuals without a formal diagnosis or standard diagnostic instruments always leads to some error. We used dementia classification algorithms for both HRS and NHATS that are well documented but do not perfectly assign dementia status. The BRFSS estimates relied on caregiver‐reported dementia status, which is also subject to error. Second, the NHATS estimates are based on a population 70 years of age and older. People younger than 70 living with dementia may have different caregiving needs, so this limited age range may lead to biased results. Third, the BRFSS data were highly processed, as they were converted from a bucketed number of hours that is reported per caregiver to a point estimate, which is reported per prevalent case of dementia. However, we incorporated uncertainty at each step via bootstrapping and using prevalence draws. Fourth, our model of the fraction of caregiving hours that are attributable to dementia was created at the national level. Therefore, an assumption was made that this fraction did not vary significantly by state, and we applied the same attributable fraction to all state estimates. This is a strong assumption, given that the overall population health and number of comorbidities do vary by state. This simplification likely led to an overattribution of care for dementia to states with worse health and vice versa. Finally, we did not have data available to incorporate dementia severity into the cost estimates.

These findings underscore the significant variation in the amount of informal care per person living with dementia that is being provided at the state level. Providing informal care can impose financial and emotional strain on families,^31^, ^32^ and this burden is not distributed evenly throughout the United States. The states with the lowest income per capita had the highest burden of informal care. An important implication for policymakers is that the U.S. health care system is heavily reliant on informal care, even as demographic shifts are expected to lead to fewer caregivers per person living with dementia. Policymakers should consider investing in programs to support informal caregivers. Given the critical role that informal caregivers play in dementia care, efforts should be made to ensure that clinicians are aware of existing resources for caregivers. Future research should investigate the relationship between caregiving and direct health spending on dementia to assess the possible trade‐offs between these types of care and to explore the potential drivers of the substantial indirect cost difference between states, such as the age structure of the caregiver population, the availability of adult day care centers and home health aides, and Medicaid support for home‐ and community‐based services.

## CONFLICT OF INTEREST STATEMENT

The authors declare no conflicts of interest.

## CONSENT STATEMENT

Consent from human subjects was not necessary for this research. All data used were secondary, de‐identified data.

## Supporting information

Supporting Information

Supporting Information
