# Supplementary material for: U.S. dementia care spending by state: 2010–2019
Source: Alzheimers Dement. 2024 Feb 27;20(4):2742–51. doi: 10.1002/alz.13746 (PMC11032574; doi:10.1002/alz.13746)
Supplement: Supplementary file 1 — Supporting Information [file ALZ-20-2742-s002.docx]

Appendix: US dementia care spending by state, 2010–2019

**Authors:** Amy Lastuka, PhD^1^, Michael R. Breshock, MSE^1^, Theresa A. McHugh PhD^1^, William T. Sogge, BA^1^, Vivianne Swart, MPH^1^, Joseph L. Dieleman, PhD^1^

**Affiliations:** ^1^Institute for Health Metrics and Evaluation, Seattle, Washington

**Corresponding author:**

Joseph L. Dieleman

Institute for Health Metrics and Evaluation

Hans Rosling Center for Population Health

3980 15^th^ Ave. NE

Seattle, WA 98195

[dieleman@uw.edu](mailto:dieleman@uw.edu)

10/30/2023

# Keywords: dementia, informal care, Alzheimer’s disease, cost, cost-of-illness study, economics, United States

# 1.0 Data

## 1.1 Data Sources

**Figure S1**. Data sources used in analysis. HRS and NHATS are filtered to people living with dementia, and BRFSS is filtered to people caring for someone with dementia. Sample size represents number of survey respondents after filtering.


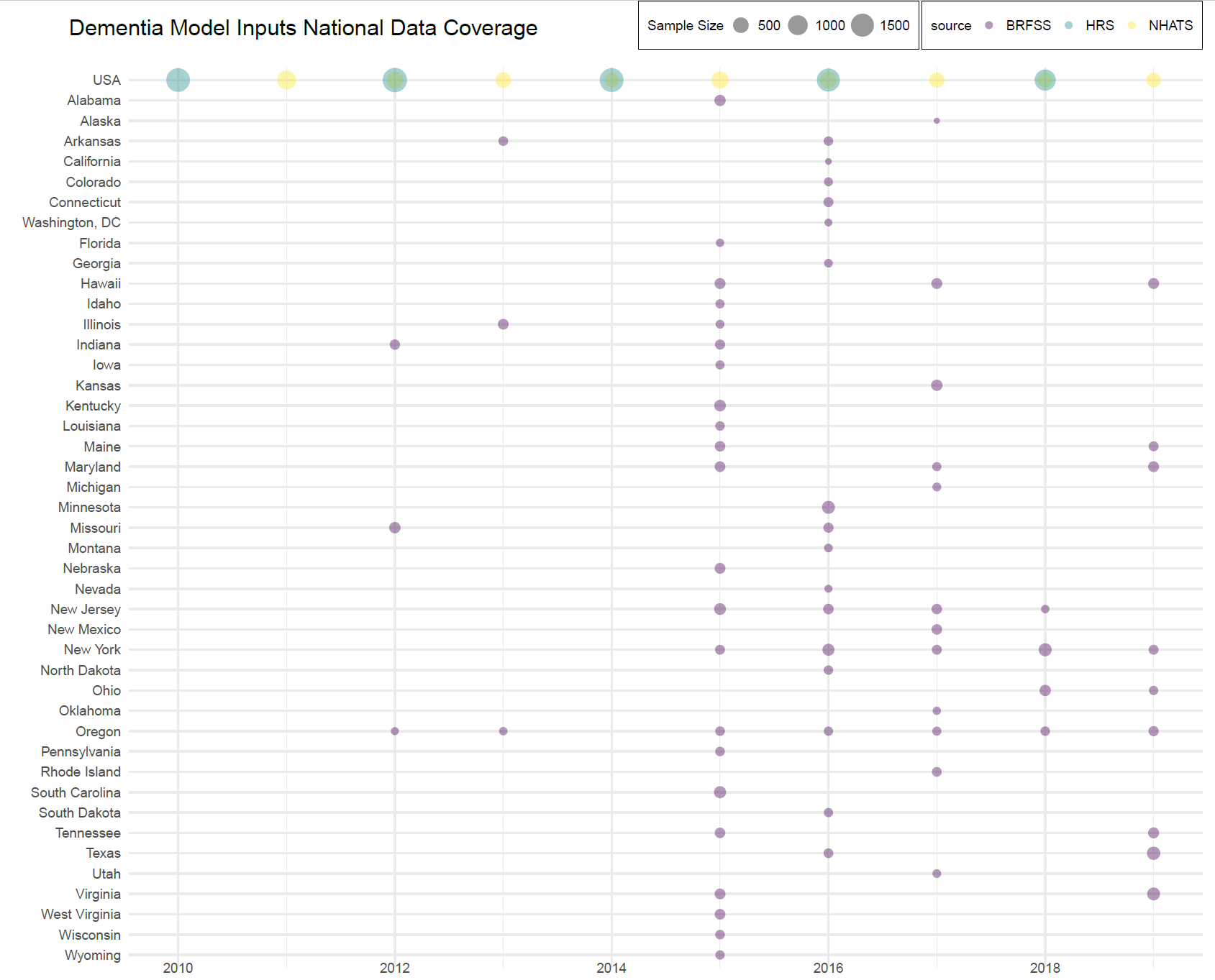


**Table S1.** Data sources used in analysis.

| **Entity** | **Source** | **Year(s) available** |
| --- | --- | --- |
| State-level caregiving hours | Behavioral Risk Factor Surveillance System^1-14^ | 2012-2013;2015-2019 |
| National caregiving hours | Health and Retirement Study^15-19^ | 2010;2012;2014;2016;2018 |
| National caregiving hours | National Health and Aging Trends Study^149-157^ | 2011-2019 |
| State-level diabetes prevalence | IHME^163^ | 2010-2019 |
| State-level years of education | IHME^164^ | 2010-2019 |
| State-level median income | Census^158^ | 2010-2019 |
| Caregiving activity type fractions | GERAS-US^159^ | 2016-2017 |
| Home Health Aide Wages | Bureau of Labor Statistics^139-148^ | 2010-2019 |
| Consumer cost of home health aide | Genworth Cost of Care Survey^160-162^ | 2012;2015;2018 |
| Average state-level wages by sex, age, and education level | Current Population Survey^20-138^ | 2010-2019 |
| Average state-level labor force participation by sex, age, and education level | Current Population Survey^20-138^ | 2010-2019 |
| State-level dementia prevalence | Global Burden of Disease^156^ | 2010-2019 |

## 1.2 GATHER Checklist.

| # | | GATHER checklist item | Description of compliance | Reference |
| --- | --- | --- | --- | --- |
| Objectives and funding | | | |  |
| 1 | | Define the indicators, populations, and time periods for which estimates were made. | Narrative provided in paper and methods appendix describing indicators, definitions, and populations | Main text (Methods) and methods appendix |
| 2 | | List the funding sources for the work. | Funding sources listed in paper | Main text (Acknowledgments) |
| Data Inputs | | | |  |
| *For all data inputs from multiple sources that are synthesized as part of the study:* | | | |  |
| 3 | | Describe how the data were identified and how the data were accessed. | Narrative provided in paper and methods appendix describing data-seeking methods | Main text (Methods) and methods appendix |
| 4 | | Specify the inclusion and exclusion criteria. Identify all ad-hoc exclusions. | Narrative provided in paper and methods appendix describing inclusion and exclusion criteria | Main text (Methods) and methods appendix |
| 5 | | Provide information on all included data sources and their main characteristics. For each data source used, report reference information or contact name/institution, population represented, data collection method, year(s) of data collection, sex and age range, diagnostic criteria or measurement method, and sample size, as relevant. | Metadata for data sources by component, geography, cause, risk, or impairment is available through an interactive, online data record | Link to the GHDx to be provided upon publication. |
| 6 | | Identify and describe any categories of input data that have potentially important biases (e.g., based on characteristics listed in item 5). | Summary of known biases included in paper narrative | Main text (Limitations) |
| *For data inputs that contribute to the analysis but were not synthesized as part of the study:* | | | |  |
| 7 | | Describe and give sources for any other data inputs. | Will be included in GHDx link | Link to the GHDx to be provided upon publication. |
| *For all data inputs:* | | | |  |
| 8 | | Provide all data inputs in a file format from which data can be efficiently extracted (e.g., a spreadsheet as opposed to a PDF), including all relevant meta-data listed in item 5. For any data inputs that cannot be shared due to ethical or legal reasons, such as third-party ownership, provide a contact name or the name of the institution that retains the right to the data. | Downloads of input data available through online tools such as the Global Health Data Exchange website;online tools, including data visualization tools; input data not available in tools will be made available upon request. Contacts and institution names will be provided for any input data that could not be shared. | The Global Health Data Exchange, [http://ghdx.healthdata.org](http://ghdx.healthdata.org/) |
| Data analysis | | | | |
| 9 | Provide a conceptual overview of the data analysis method. A diagram may be helpful. | | Flow diagrams, write ups of the overall methodological processes, as well as specific modeling processes, have been provided | Main text (Methods) and methods appendix |
| 10 | Provide a detailed description of all steps of the analysis, including mathematical formulae. This description should cover, as relevant, data cleaning, data pre-processing, data adjustments and weighting of data sources, and mathematical or statistical model(s). | | Flow diagrams and corresponding methodological write-ups have been provided | Main text (Methods) and methods appendix |
| 11 | Describe how candidate models were evaluated and how the final model(s) were selected. | | Details on evaluation of model performance have been provided | Methods appendix |
| 12 | Provide the results of an evaluation of model performance, if done, as well as the results of any relevant sensitivity analysis. | | Details on evaluation of model performance have been provided | Main text (Methods – 3.2 Sensitivity Analyses) and methods appendix |
| 13 | Describe methods for calculating uncertainty of the estimates. State which sources of uncertainty were, and were not, accounted for in the uncertainty analysis. | | Details on uncertainty calculations have been provided | Methods appendix |
| 14 | State how analytic or statistical source code used to generate estimates can be accessed. | | Access statement provided | Code is provided in an online repository, link to the GHDx to be provided upon publication. |
| Results and Discussion | | | | |
| 15 | Provide published estimates in a file format from which data can be efficiently extracted. | | Results are available through the Global Health Data Exchange | Link to the GHDx to be provided upon publication. |
| 16 | Report a quantitative measure of the uncertainty of the estimates (e.g. uncertainty intervals). | | Uncertainty intervals are provided for final estimates | Main text, methods appendix, and online data tools (the Global Health Data Exchange, link to the GHDx to be provided upon publication.) |
| 17 | Interpret results in light of existing evidence. If updating a previous set of estimates, describe the reasons for changes in estimates. | | Discussion of methodological differences between our estimates and other available evidence provided in the paper and methods appendix | Main text (Methods and Discussion) and methods appendix |
| 18 | Discuss limitations of the estimates. Include a discussion of any modelling assumptions or data limitations that affect interpretation of the estimates. | | Discussion of limitations was provided | Main text (Limitations) and methods appendix |

# 2.0 Technical appendix

## 2.1 Caregiving Hours Estimate Methodology

**2.1.1 Behavioral Risk Factors Surveillance System (BRFSS)**

The Behavioral Risk Factors and Surveillance System (BRFSS) survey is conducted by individual states and offers an optional caregiver module.^157^ We collected data from 78 state-years from 2012 through 2019. The states and years used in our analysis are shown in Figure S1. In BRFSS, dementia status is based on caregiver report, and no cognitive or demographic information about the person living with dementia is provided.

The BRFSS caregiver module asks respondents how many hours of care they are providing per week, without making any distinction between type of activity (activities of daily living [ADLs], instrumental activities of daily living [IADLs], or other care, often labeled supervision). In 2012 and 2013, the caregiver module asked about weekly hours of caregiving with a continuous response option (0–168 hours per week), while later years switched to a bucketed response option. We used the responses from 2012 and 2013 to create a distribution for each bucket range and then sampled from that distribution to transform the bucketed responses to point estimates. We repeated the sampling 1000 times to bootstrap confidence intervals for these point estimates. Prior to 2019, the survey respondents were asked about the primary health condition of the care recipient, with “Alzheimer’s Disease or dementia” being one of the response options. Starting in 2019, a question was added to ask respondents whether the care recipient has Alzheimer's Disease or dementia. On average, across all 10 states that administered the caregiver module in 2019, using the more inclusive question led to an estimate of 2.2 times more caregivers reporting providing care for someone with Alzheimer’s Disease or dementia. Therefore, to estimate care hours per prevalent case of dementia, we scaled up the total number of care hours provided by 2.2 to account for all dementia caregivers, regardless of whether dementia was reported as the primary condition of the care recipient. We then divided total caregiving hours by the dementia prevalence extracted from the Global Burden of Disease^158^ to calculate hours per prevalent case of dementia. To incorporate uncertainty in the dementia prevalence estimates, we extracted 1000 independent draws of dementia prevalence estimates from the Global Burden of Disease and completed our analysis 1000 independent times.^159^

**2.1.2 National Health and Aging Trends Study**

The National Health and Aging Trends Study (NHATS) is a nationally representative panel study of Medicare recipients aged 65 and older. NHATS contains detailed information about each survey respondent’s cognition, comorbidities, and care needs. We first classified respondents as having probable dementia or not, following the criteria described in Kasper et. al.^160^ We then calculated a national average of caregiving hours per prevalent case of dementia for each year from 2011 to 2019. Survey respondents under the age of 70 years were excluded from our analysis due to the NHATS panel design. Survey respondents are followed over time so that the minimum age represented in the survey increases to 69 before additional respondents are recruited.

**2.1.3 Health and Retirement Study**

The Health and Retirement Study (HRS) is a nationally representative survey of U.S. adults aged 50 years and older. Survey respondents under the age of 55 years were excluded from our analysis due to the aging cohort structure of HRS. We used the Langa-Weir classification^161^ of cognitive function to determine dementia status among survey respondents.

The HRS questionnaire asks survey respondents about help that they receive for a series of activities corresponding to ADLs and IADLs. The measure of weekly caregiving time per prevalent case of dementia estimated from HRS data is therefore smaller than estimates from NHATS and BRFSS, which are inclusive of supervisory time (e.g. miscellaneous care that does not fall into the categories of ADL or IADL support). This offset is accounted for when the caregiving estimates from these three sources are combined into a linear model.

## 2.2 Replacement Cost Model

**2.2.1 Outlier analysis for replacement cost model**

When subset to caregivers who are caring for someone with dementia or Alzheimer’s disease, the sample size became very small in some states. To identify outliers and eliminate implausible weekly caregiving hours estimates, we calculated a modified z-score for all state estimates. Table S2 shows the outliers that were eliminated for the caregiving hours model top-coded to 112 hours per week.

**Table S2.** Outliers based on modified z-score greater than 2. Caregiving hours top-coded to 112 per week.

| State | Year | Average weekly caregiving hours per case | Sample size |
| --- | --- | --- | --- |
| Arizona | 2016 | 67.0 | 92 |
| Georgia | 2012 | 73.9 | 136 |
| Iowa | 2012 | 13.7 | 64 |
| Maine | 2012 | 13.5 | 130 |
| Mississippi | 2015 | 84.2 | 131 |
| Tennessee | 2016 | 67.4 | 140 |
| Utah | 2015 | 71.3 | 76 |
| Utah | 2019 | 71.4 | 132 |

**2.2.2 Model selection for replacement cost model**

For the linear model of caregiving hours, several variables that were plausibly relevant to caregiving hours were tested. Each variable was regressed independently on logged caregiving hours. All variables that were significantly correlated with caregiving hours were combined into one multivariate model. Finally, variables that were not statistically significant in the multivariate model were pruned to create the final model specification. Pruning did not have a large effect on the model fit, so the more parsimonious model was selected. The same selection process was used for the linear model of expected wages. Table S3 shows the model results for the linear model of caregiving hours top-coded to 112 hours per week.

**Table S3**. Linear model of caregiving hours top-coded to 112 hours per week
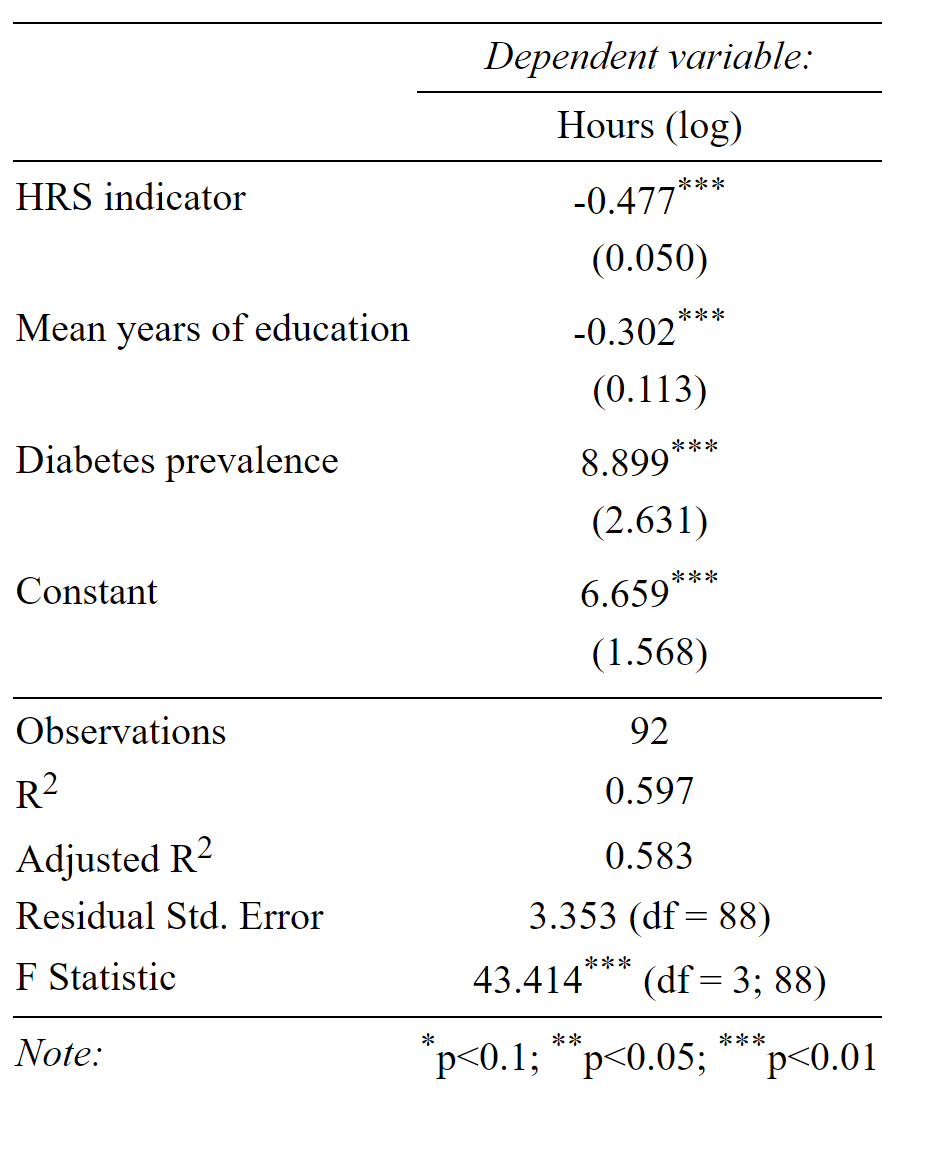


## 2.3 Forgone Wage Model

**2.3.1 Outlier analysis for forgone wage model**

When subset to caregivers who are caring for someone with dementia or Alzheimer’s disease, the sample size became very small in some states. To identify outliers and eliminate implausible weekly caregiving hours estimates, we calculated a modified z-score for all state estimates. Table S4 shows the outliers that were eliminated for the caregiving hours model top-coded to 40 hours per week. Table S5 shows the model results for the linear model of caregiving hours top-coded to 40 hours per week.

Table S6 shows the outliers that were eliminated for the expected caregiver wage model. Table S7 shows the model results for the linear model of caregiver wage. Figures S2 and S3 show results from the replacement cost and forgone wages models in which no outliers had been removed.

**Table S4.** Outliers for caregiving hours based on modified z-score greater than 2.5. Caregiving hours top-coded to 40 per week.

| State | Year | Average weekly caregiving hours per case | Sample size |
| --- | --- | --- | --- |
| Georgia | 2012 | 23.6 | 136 |
| Mississippi | 2015 | 23.4 | 131 |
| United States | 2013 | 25.0 | 506 |
| United States | 2014 | 23.4 | 387 |
| United States | 2016 | 24.1 | 634 |

**Table S5.** Linear model of caregiving hours top-coded to 40 hours per week


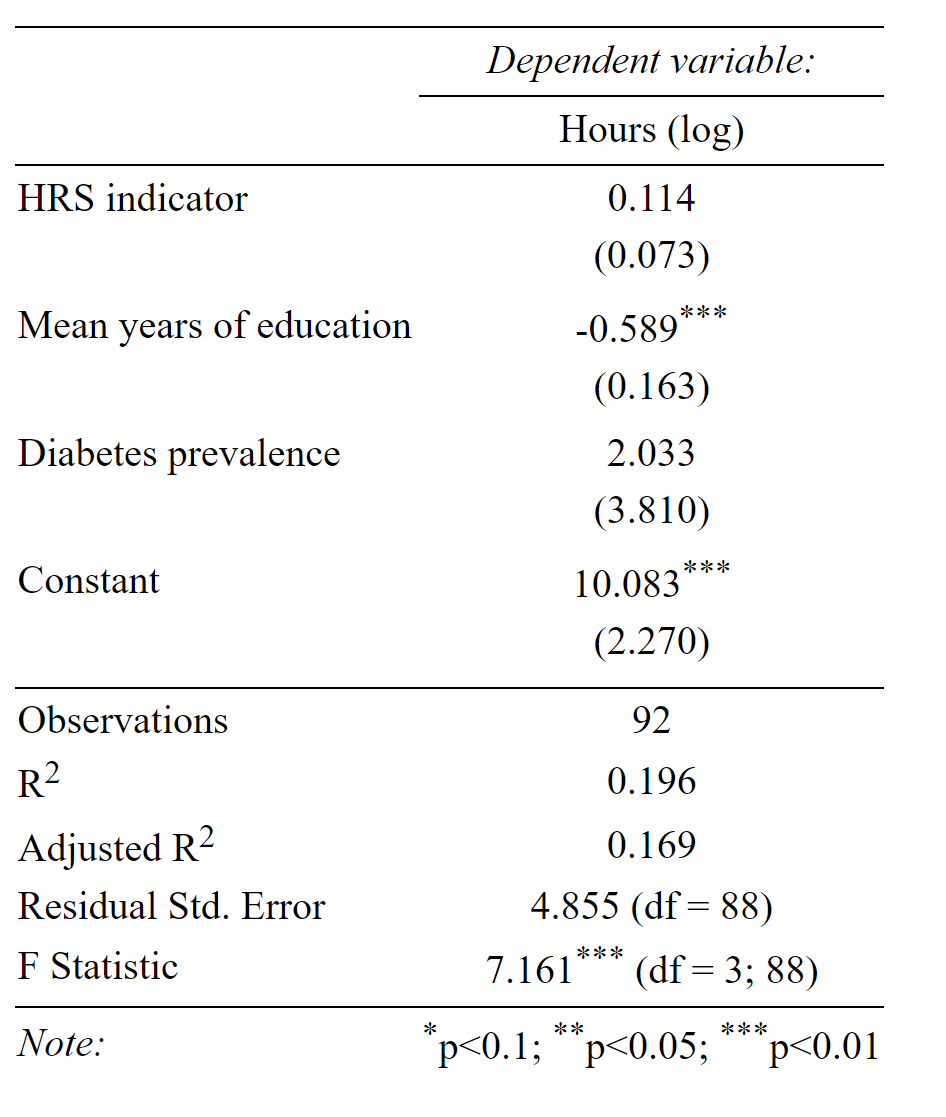


**Table S6**. Outliers for expected wage model based on modified z-score greater than 2.

| State | Year | Expected hourly wage | Sample size |
| --- | --- | --- | --- |
| Connecticut | 2016 | 19.10 | 113 |
| Maryland | 2015 | 17.51 | 141 |
| Maryland | 2017 | 17.66 | 81 |
| Maryland | 2019 | 18.51 | 150 |
| Montana | 2016 | 8.31 | 68 |
| Nevada | 2016 | 8.58 | 49 |
| New Jersey | 2015 | 17.54 | 200 |
| Rhode Island | 2017 | 19.62 | 102 |

**Table S7.** Linear model of expected caregiver wage


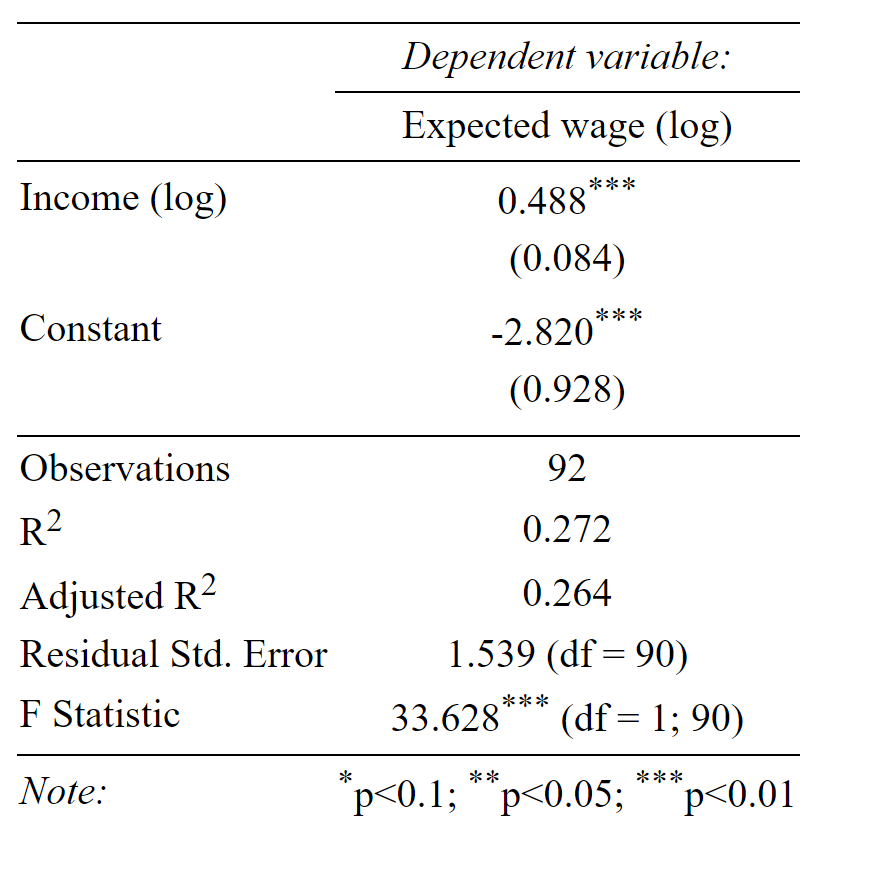


**Figure S2**. Map of replacement cost and forgone wage cost per prevalent case of dementia with outlier state data included in model
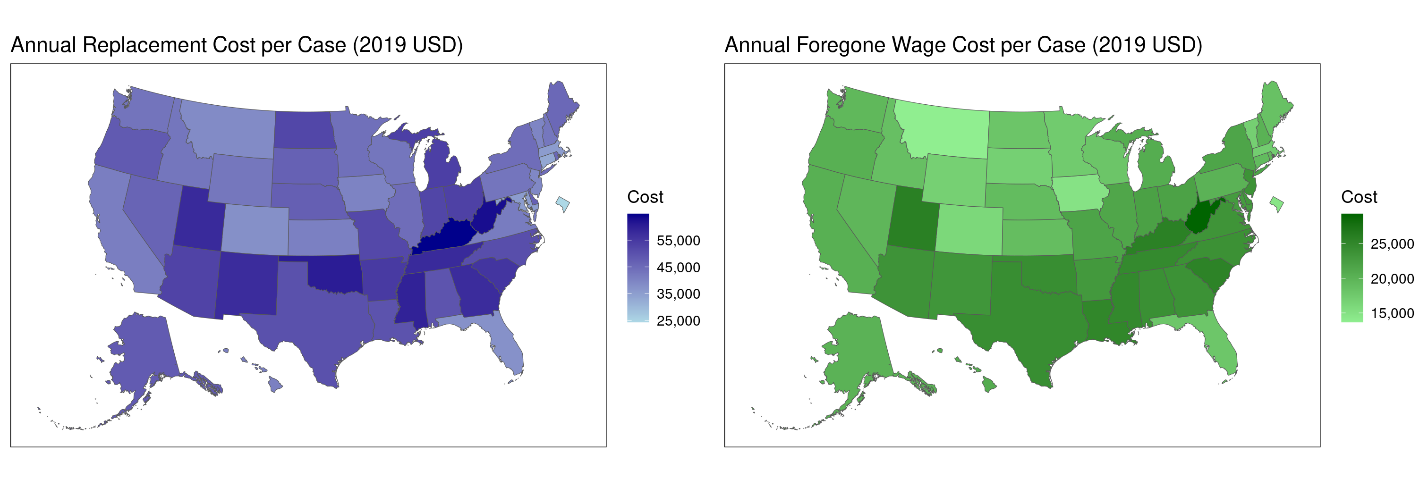

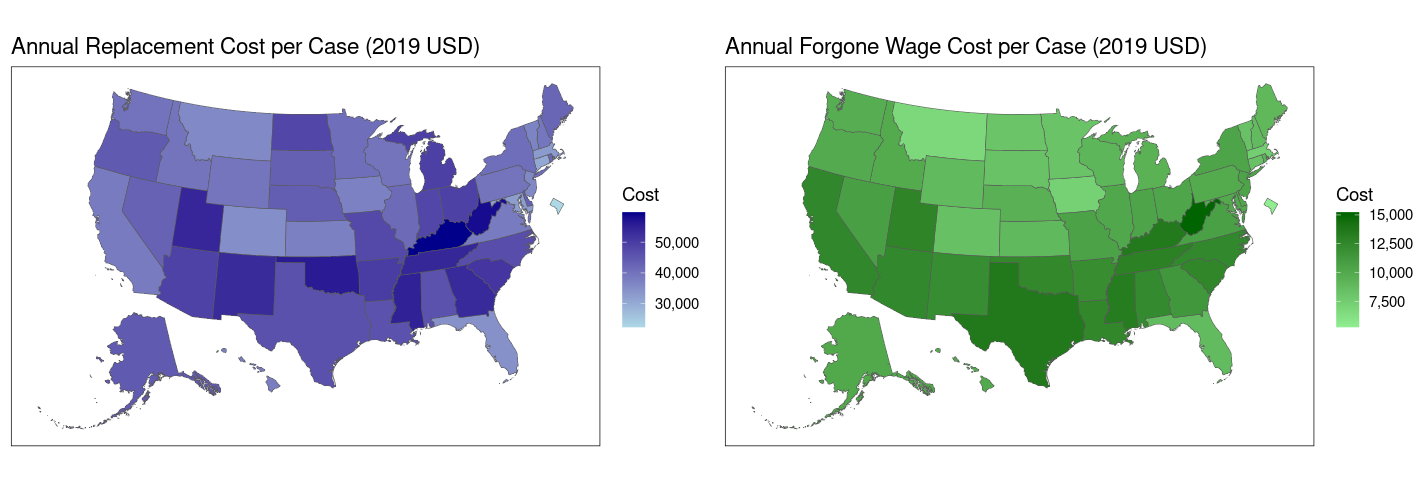


**Figure S3**. Costs per prevalent case from 2010 through 2019 with outlier state data included in model


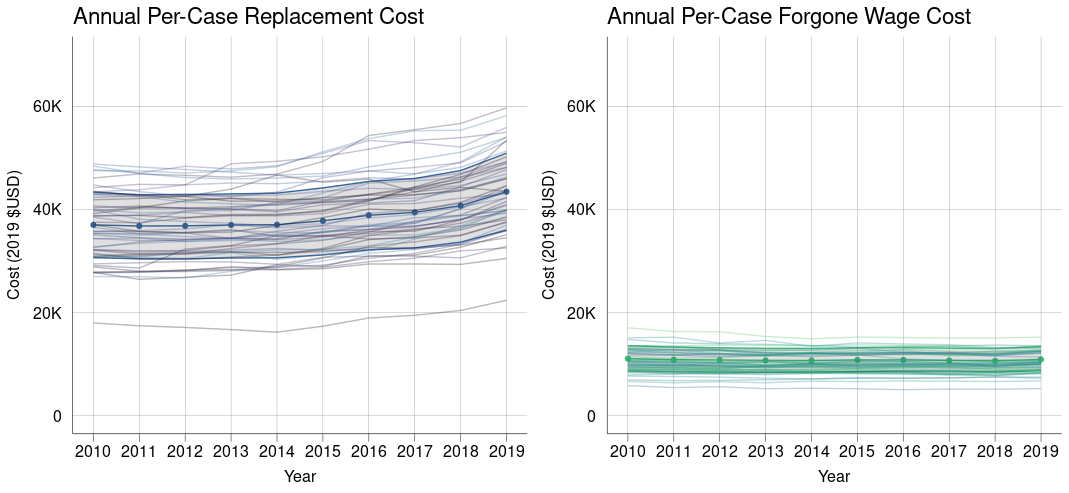


## 2.4 Attributable Fraction Model

The attributable fraction was modeled using individual-level data from NHATS Rounds 1-9. The sample included only people classified with dementia. The NHATS survey includes questions about the sample person’s health conditions and demographics, however not all respondents answered every question resulting in missing values in the sample data. The Amelia package^162^ was used to fill in these missing values with multiple imputation. The variables that were imputed were patient age, sex, race/ethnicity, heart disease, high blood pressure, arthritis, diabetes, lung disease, stroke, cancer, anxiety and depression. We also considered patient marital status and number of children as variables to include in the model but decided to exclude them as they were insignificant.

The attributable fraction was modeled using dependent variable log weekly care time and independent variables depression, high blood pressure, and stroke (see Table S8). These independent variables were selected from a larger list of conditions including diabetes, heart disease, arthritis, cancer, lung disease, and anxiety. The conditions included in the final model were chosen using Bayesian model selection with the BAS package^163^. Conditions that were not found to be significant independent variables by the model selection were excluded.

**Table S8.** Output from dementia attribution model

| **Condition** | **Attributable Fraction** |
| --- | --- |
| **Dementia** | **0.720** |
| Depression | 0.076 |
| High blood pressure | 0.190 |
| Stroke | 0.014 |

## 2.5 Home Health Aide Wages

We extracted the average home health aide wage by state and year from the US Bureau of Labor Statistics (BLS) Occupational Employment and Wage Statistics (OEWS) program. This data provides wage and employment statistics for an exhaustive list of occupations. Data from 2010-2018 had two separate occupational categories for “Home Health Aides” and “Personal Care Aides.” In some states and years data could be released because of quality or confidentiality reasons. Sometimes only one or a few variables of data are missing. All missing values were interpolated. From 2019 onward, these categories were combined into a single “Home Health and Personal Care Aides” category. In order to accurately compare data across time, the “Home Health Aides” and “Personal Care Aides” categories present from 2010-2018 were combined into a singular “Home Health and Personal Care Aides” category.

To convert home health aide wages into costs, we used the Genworth Cost of Care survey to calculate an average markup by dividing Genworth home health aide costs by BLS home health aide wages. The Genworth cost data is only available for 2012, 2015, 2018, and 2021. The missing years were interpolated using a linear regression model to estimate a cost markup ratio for each year included in our analysis. Once the wage data was converted to cost, the final home health aide costs were price parity adjusted and converted to 2019 $USD.

## 2.6 Expected Caregiver Wages

For the forgone wage approach, we imputed each caregiver’s wage using data from the Basic Monthly Current Population Survey (CPS). We divided age into six groups: 18­­–34, 35–44, 45–54, 55–64, 65–74, and 75 and up. We grouped educational attainment into three categories: high school or less, some college, and Bachelor’s degree or more. The average wage and labor force participation for each demographic group (age, education, and sex) was calculated for each state and year. The average wage for all subsets of demographic groups (e.g. age and sex) were also calculated so that if a caregiver was missing any demographic information their wage could be imputed based on the demographic information available.

## 2.7 State level estimates

Table S9 shows the state-level estimates for the replacement cost model and the forgone wage model in 2019, along with 95% confidence intervals.

**Table S9. US Per-Case Cost Estimates 2019 ($USD)**

| **State** | **Replacement Cost** | **Cost of Forgone Wages** |
| --- | --- | --- |
| US National | 42,422 (35,422 – 49,181) | 10,677 (8,611 – 12,904) |
| Alabama | 43,641 (36,227 – 50,878) | 12,441 (10,052 – 15,008) |
| Alaska | 44,547 (36,760 – 51,830) | 9,766 (7,876 – 11,813) |
| Arizona | 43,994 (36,618 – 51,704) | 12,120 (9,788 – 14,636) |
| Arkansas | 48,932 (40,863 – 57,290) | 11,984 (9,681 – 14,462) |
| California | 38,613 (32,108 – 45,328) | 11,442 (9,232 – 13,827) |
| Colorado | 36,256 (30,006 – 42,503) | 8,182 (6,594 – 9,910) |
| Connecticut | 30,459 (25,410 – 35,912) | 7,953 (6,408 – 9,641) |
| Delaware | 41,508 (34,524 – 48,546) | 10,448 (8,427 – 12,631) |
| District of Columbia | 22,436 (18,705 – 26,214) | 5,534 (4,451 – 6,735) |
| Florida | 33,490 (27,922 – 39,163) | 8,808 (7,103 – 10,655) |
| Georgia | 49,374 (40,611 – 58,785) | 11,066 (8,935 – 13,367) |
| Hawaii | 38,533 (31,923 – 45,148) | 9,834 (7,928 – 11,899) |
| Idaho | 39,685 (33,236 – 46,177) | 9,590 (7,734 – 11,597) |
| Illinois | 41,165 (33,980 – 47,829) | 10,208 (8,232 – 12,347) |
| Indiana | 45,741 (38,076 – 53,214) | 10,336 (8,337 – 12,493) |
| Iowa | 40,617 (33,718 – 47,386) | 7,185 (5,789 – 8,710) |
| Kansas | 36,840 (30,685 – 42,946) | 8,871 (7,152 – 10,736) |
| Kentucky | 56,684 (47,043 – 66,416) | 13,502 (10,913 – 16,277) |
| Louisiana | 43,609 (36,037 – 50,985) | 12,944 (10,461 – 15,607) |
| Maine | 46,020 (38,012 – 53,927) | 8,998 (7,255 – 10,886) |
| Maryland | 32,205 (26,746 – 37,766) | 9,274 (7,476 – 11,229) |
| Massachusetts | 33,238 (27,345 – 38,715) | 7,420 (5,977 – 9,000) |
| Michigan | 47,549 (39,490 – 54,971) | 9,679 (7,806 – 11,701) |
| Minnesota | 41,171 (34,416 – 47,898) | 8,095 (6,523 – 9,812) |
| Mississippi | 47,644 (39,476 – 55,652) | 12,617 (10,198 – 15,209) |
| Missouri | 46,722 (38,819 – 54,606) | 10,775 (8,694 – 13,021) |
| Montana | 36,060 (29,923 – 42,290) | 7,230 (5,826 – 8,762) |
| Nebraska | 43,167 (35,589 – 50,393) | 9,341 (7,531 – 11,303) |
| Nevada | 42,666 (35,554 – 50,102) | 10,915 (8,806 – 13,190) |
| New Hampshire | 38,683 (32,004 – 45,080) | 8,722 (7,030 – 10,564) |
| New Jersey | 36,022 (29,904 – 42,048) | 10,294 (8,301 – 12,453) |
| New Mexico | 51,690 (42,902 – 60,477) | 12,233 (9,884 – 14,758) |
| New York | 40,870 (34,081 – 47,863) | 10,483 (8,455 – 12,674) |
| North Carolina | 44,828 (37,086 – 52,204) | 12,342 (9,970 – 14,895) |
| North Dakota | 47,661 (39,427 – 55,733) | 8,240 (6,642 – 9,977) |
| Ohio | 47,503 (39,830 – 55,587) | 10,473 (8,451 – 12,657) |
| Oklahoma | 53,909 (44,874 – 62,958) | 12,437 (10,048 – 15,006) |
| Oregon | 44,774 (37,396 – 51,897) | 9,945 (8,020 – 12,027) |
| Pennsylvania | 39,371 (32,621 – 46,182) | 9,814 (7,914 – 11,871) |
| Rhode Island | 42,062 (34,600 – 49,013) | 8,580 (6,917 – 10,387) |
| South Carolina | 49,184 (40,531 – 57,832) | 12,624 (10,198 – 15,233) |
| South Dakota | 42,998 (35,758 – 50,269) | 8,215 (6,622 – 9,946) |
| Tennessee | 42,998 (35,758 – 50,269) | 13,134 (10,616 – 15,833) |
| Texas | 45,562 (37,547 – 53,283) | 13,077 (10,565 – 15,775) |
| Utah | 44,745 (37,141 – 52,245) | 12,810 (10,346 – 15,465) |
| Vermont | 37,594 (30,838 – 44,702) | 8,188 (6,599 – 9,918) |
| Virginia | 38,356 (31,976 – 44,554) | 10,837 (8,740 – 13,102) |
| Washington | 40,111 (33,326 – 47,300) | 9,313 (7,508 – 11,270) |
| West Virginia | 54,154 (45,131 – 62,954) | 15,297 (12,372 – 18,417) |
| Wisconsin | 40,103 (33,208 – 46,743) | 8,964 (7,226 – 10,849) |
| Wyoming | 40,636 (33,377 – 47,896) | 8,634 (6,961 – 10,447) |

## 2.8 Projected costs

Table S10 shows the projected costs for the low-growth scenario in which per-case costs of informal care stay the same and growth is driven by an increase in dementia prevalence. Table S11 shows the projected costs for the high-growth scenario in which per-cases costs of informal care continue to increase linearly based on their past trends.

**Table S10**. Low-growth scenario: current and projected costs of informal dementia care

| Total cost (billions of $) | | |
| --- | --- | --- |
|  | Replacement cost | Forgone wage cost |
| 2019 | 230 (189 – 271) | 58 (46 – 71) |
| 2030 | 276 (254 – 303) | 75 (69 – 82) |
| 2040 | 358 (324 – 397) | 97 (88 – 108) |
| 2050 | 404 (354 – 455) | 110 (96 – 124) |
| Per-capita cost ($) | | |
|  | Replacement cost | Forgone wage cost |
| 2019 | 933 (769 – 1,099) | 235 (188 – 288) |
| 2030 | 1,042 (893 – 1,211) | 283 (243 – 329) |
| 2040 | 1,309 (1,103 – 1,537) | 356 (300 – 418) |
| 2050 | 1,468 (1,199 – 1,778) | 399 (326 – 483) |

Per-capita costs are total costs divided by the number of people aged 20 and older.

**Table S11**. High-growth scenario: current and projected costs of informal dementia care

| Total cost (billions of $) | | |
| --- | --- | --- |
|  | Replacement cost | Forgone wage cost |
| 2019 | 230 (189 – 271) | 58 (46 – 71) |
| 2030 | 330 (265 – 403) | 77 (55 – 101) |
| 2040 | 474 (365 – 609) | 100 (63 – 144) |
| 2050 | 586 (430 – 774) | 115 (59 – 179) |
| Per-capita cost ($) | | |
|  | Replacement cost | Forgone wage cost |
| 2019 | 933 (769 – 1,099) | 235 (188 – 288) |
| 2030 | 1,247 (950 – 1,594) | 289 (207 – 391) |
| 2040 | 1,734 (1,256 – 2,291) | 368 (224 – 549) |
| 2050 | 2,131 (1,473 – 2,915) | 418 (205 – 662) |

Per-capita costs are total costs divided by the number of people aged 20 and older.

## 2.9 Forgone Wage Cost Decomposition

Figure S4 shows the variation in per capita forgone wage costs by comparing each state’s cost to that in Alaska, the state with the lowest per capita forgone wage cost; it also identifies the contributions of age profile, dementia prevalence, expected caregiver wage, and hours of informal care per prevalent case to the cost difference.

**Figure S4**. Das Gupta decomposition of per-capita forgone wage costs. Costs are relative to the per-capita cost in Alaska.


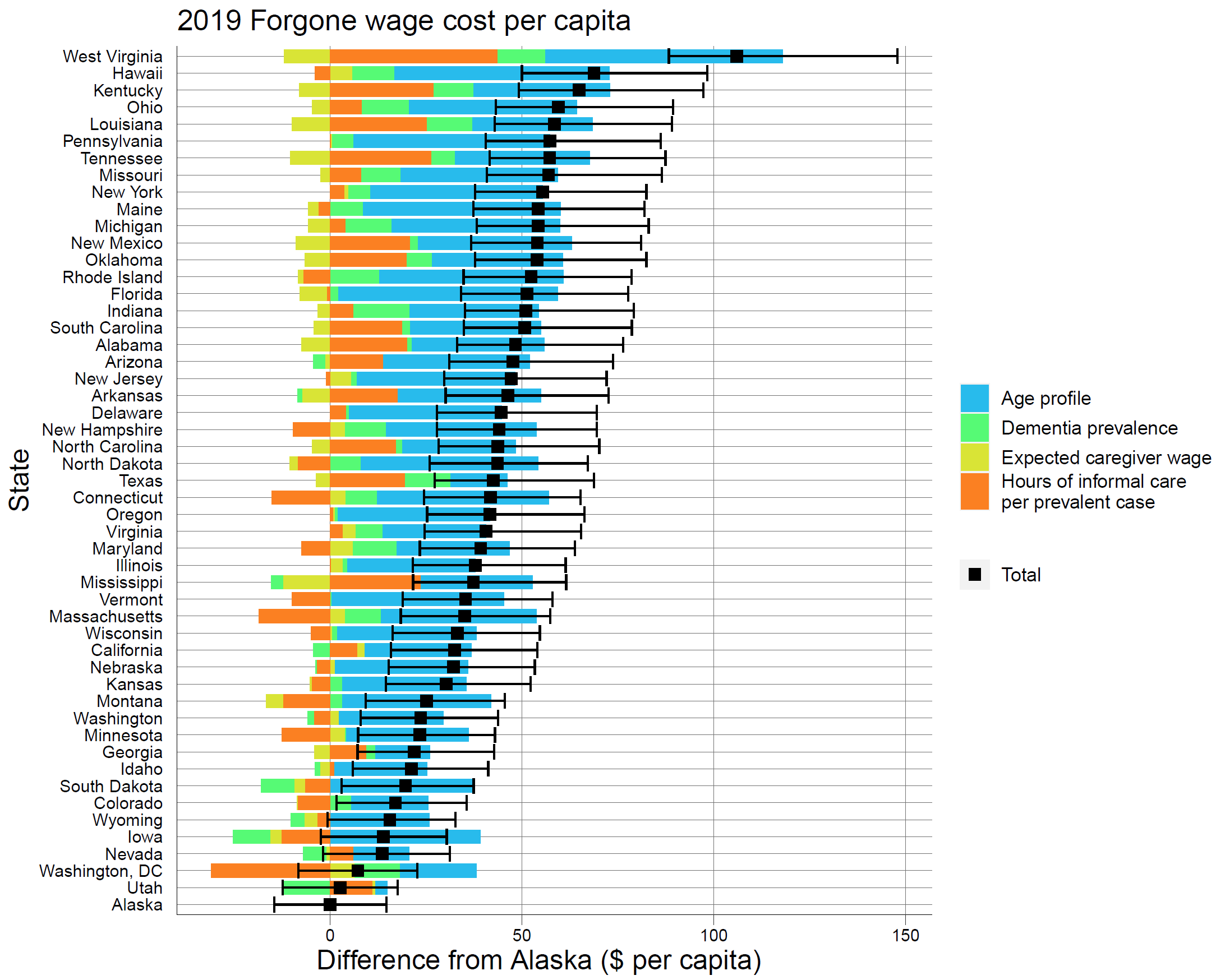


## 2.10 Sensitivity analysis

As a sensitivity analysis, we report results for a forgone wage model that allows weekly hours of up to 112 per week. Table S12 shows a comparison of the two specifications of the forgone wage model. Table S13 shows projected costs for the forgone wage model top-coded to 112 hours per week.

**Table S12.** Comparison of US Per-Case Cost Estimates 2019 ($USD) for Forgone Wages Top-Coded to 112 Hours per Week and 40 Hours per Week

| **State** | **Forgone Wages (112 Hours)** | **Forgone Wages (40 Hours)** |
| --- | --- | --- |
| US National | 20,786 (17,122 – 24,363) | 10,677 (8,611 – 12,904) |
| Alabama | 22,912 (18,879 – 26,845) | 12,441 (10,052 – 15,008) |
| Alaska | 19,652 (16,191 – 23,029) | 9,766 (7,876 – 11,813) |
| Arizona | 20,953 (17,264 – 24,552) | 12,120 (9,788 – 14,636) |
| Arkansas | 22,530 (18,564 – 26,398) | 11,984 (9,681 – 14,462) |
| California | 20,034 (16,506 – 23,477) | 11,442 (9,232 – 13,827) |
| Colorado | 16,216 (13,360 – 19,004) | 8,182 (6,594 – 9,910) |
| Connecticut | 17,756 (14,628 – 20,808) | 7,953 (6,408 – 9,641) |
| Delaware | 21,758 (17,927 – 25,495) | 10,448 (8,427 – 12,631) |
| District of Columbia | 13,983 (11,519 – 16,389) | 5,534 (4,451 – 6,735) |
| Florida | 17,326 (14,275 – 20,303) | 8,808 (7,103 – 10,655) |
| Georgia | 22,140 (18,242 – 25,941) | 11,066 (8,935 – 13,367) |
| Hawaii | 20,580 (16,956 – 24,117) | 9,834 (7,928 – 11,899) |
| Idaho | 18,348 (15,117 – 21,501) | 9,590 (7,734 – 11,597) |
| Illinois | 20,888 (17,209 – 24,477) | 10,208 (8,232 – 12,347) |
| Indiana | 20,728 (17,078 – 24,288) | 10,336 (8,337 – 12,493) |
| Iowa | 15,864 (13,070 – 18,591) | 7,185 (5,789 – 8,710) |
| Kansas | 18,166 (14,967 – 21,288) | 8,871 (7,152 – 10,736) |
| Kentucky | 24,628 (20,293 – 28,855) | 13,502 (10,913 – 16,277) |
| Louisiana | 24,125 (19,878 – 28,265) | 12,944 (10,461 – 15,607) |
| Maine | 19,903 (16,398 – 23,322) | 8,998 (7,255 – 10,886) |
| Maryland | 21,230 (17,491 – 24,880) | 9,274 (7,476 – 11,229) |
| Massachusetts | 16,702 (13,760 – 19,574) | 7,420 (5,977 – 9,000) |
| Michigan | 19,872 (16,373 – 23,286) | 9,679 (7,806 – 11,701) |
| Minnesota | 17,030 (14,030 – 19,958) | 8,095 (6,523 – 9,812) |
| Mississippi | 22,101 (18,211 – 25,894) | 12,617 (10,198 – 15,209) |
| Missouri | 20,399 (16,807 – 23,902) | 10,775 (8,694 – 13,021) |
| Montana | 14,730 (12,135 – 17,262) | 7,230 (5,826 – 8,762) |
| Nebraska | 18,425 (15,180 – 21,592) | 9,341 (7,531 – 11,303) |
| Nevada | 19,168 (15,793 – 22,461) | 10,915 (8,806 – 13,190) |
| New Hampshire | 19,179 (15,801 – 22,475) | 8,722 (7,030 – 10,564) |
| New Jersey | 23,039 (18,982 – 26,998) | 10,294 (8,301 – 12,453) |
| New Mexico | 22,921 (18,886 – 26,856) | 12,233 (9,884 – 14,758) |
| New York | 21,369 (17,606 – 25,040) | 10,483 (8,455 – 12,674) |
| North Carolina | 22,788 (18,776 – 26,701) | 12,342 (9,970 – 14,895) |
| North Dakota | 17,538 (14,449 – 20,552) | 8,240 (6,642 – 9,977) |
| Ohio | 21,130 (17,410 – 24,759) | 10,473 (8,451 – 12,657) |
| Oklahoma | 23,462 (19,332 – 27,490) | 12,437 (10,048 – 15,006) |
| Oregon | 20,206 (16,648 – 23,678) | 9,945 (8,020 – 12,027) |
| Pennsylvania | 19,577 (16,129 – 22,940) | 9,814 (7,914 – 11,871) |
| Rhode Island | 17,548 (14,458 – 20,564) | 8,580 (6,917 – 10,387) |
| South Carolina | 24,378 (20,086 – 28,563) | 12,624 (10,198 – 15,233) |
| South Dakota | 16,499 (13,593 – 19,335) | 8,215 (6,622 – 9,946) |
| Tennessee | 23,254 (19,161 – 27,245) | 13,134 (10,616 – 15,833) |
| Texas | 23,758 (19,575 – 27,836) | 13,077 (10,565 – 15,775) |
| Utah | 21,008 (17,309 – 24,618) | 12,810 (10,346 – 15,465) |
| Vermont | 16,832 (13,867 – 19,726) | 8,188 (6,599 – 9,918) |
| Virginia | 22,905 (18,872 – 26,840) | 10,837 (8,740 – 13,102) |
| Washington | 18,995 (15,649 – 22,259) | 9,313 (7,508 – 11,270) |
| West Virginia | 27,583 (22,729 – 32,315) | 15,297 (12,372 – 18,417) |
| Wisconsin | 17,852 (14,708 – 20,921) | 8,964 (7,226 – 10,849) |
| Wyoming | 17,026 (14,027 – 19,951) | 8,634 (6,961 – 10,447) |

**Table S13**. Cost projections for low-growth and high-growth scenarios with forgone wages top-coded to 112 hours per week.

| Total cost (billions of $) | | |
| --- | --- | --- |
|  | Low-growth scenario | High-growth scenario |
| 2019 | 113 (92 – 134) | 113 (92 – 134) |
| 2030 | 139 (128 – 153) | 162 (128 – 200) |
| 2040 | 181 (164 – 200) | 229 (170 – 297) |
| 2050 | 204 (179 – 230) | 280 (197 – 377) |
| Per-capita cost ($) | | |
|  | Low-growth scenario | High-growth scenario |
| 2019 | 457 (375 – 544) | 457 (375 – 544) |
| 2030 | 526 (450 – 611) | 612 (465 – 780) |
| 2040 | 661 (557 – 776) | 838 (598 – 1,131) |
| 2050 | 741 (605 – 897) | 1,018 (684 – 1,447) |

## References

1. Centers for Disease Control and Prevention (CDC). United States Behavioral Risk Factor Surveillance System 2015. Atlanta, United States of America: Centers for Disease Control and Prevention (CDC), 2016.

2. Centers for Disease Control and Prevention (CDC). United States Behavioral Risk Factor Surveillance System 2016. Atlanta, United States of America: Centers for Disease Control and Prevention (CDC), 2017.

3. Centers for Disease Control and Prevention (CDC). United States Behavioral Risk Factor Surveillance System 2017. Atlanta, United States of America: Centers for Disease Control and Prevention (CDC), 2018.

4. Centers for Disease Control and Prevention (CDC). United States Behavioral Risk Factor Surveillance System 2018. Atlanta, United States of America: Centers for Disease Control and Prevention (CDC), 2019

5. Centers for Disease Control and Prevention (CDC). United States Behavioral Risk Factor Surveillance System 2019. Atlanta, United States of America: Centers for Disease Control and Prevention (CDC), 2020

6. Health and Retirement Study, (Biennial 2010) public use dataset. Produced and distributed by the University of Michigan with funding from the National Institute on Aging (grant number NIA U01AG009740). Ann Arbor, MI, (2014).

7. Health and Retirement Study, (Biennial 2012) public use dataset. Produced and distributed by the University of Michigan with funding from the National Institute on Aging (grant number NIA U01AG009740). Ann Arbor, MI, (2015).

8. Health and Retirement Study, (Biennial 2014) public use dataset. Produced and distributed by the University of Michigan with funding from the National Institute on Aging (grant number NIA U01AG009740). Ann Arbor, MI, (2016).

9. "Health and Retirement Study, 2016 Core V1 Final public use dataset. Produced and distributed by the University of Michigan with funding from the National Institute on Aging (grant number NIA U01AG009740). Ann Arbor, MI, 2021.

10. Health and Retirement Study, ([insert Product Name]) public use dataset. Produced and distributed by the University of Michigan with funding from the National Institute on Aging (grant number NIA U01AG009740). Ann Arbor, MI, (year).

11. United States Census Bureau (USCB). United States Current Population Survey, January 2010. Washington, D.C., United States of America: United States Census Bureau (USCB).

12. United States Census Bureau (USCB). United States Current Population Survey, February 2010. Washington, D.C., United States of America: United States Census Bureau (USCB).

13. United States Census Bureau (USCB). United States Current Population Survey, March 2010. Washington, D.C., United States of America: United States Census Bureau (USCB).

14. United States Census Bureau (USCB). United States Current Population Survey, April 2010. Washington, D.C., United States of America: United States Census Bureau (USCB).

15. United States Census Bureau (USCB). United States Current Population Survey, May 2010. Washington, D.C., United States of America: United States Census Bureau (USCB).

16. United States Census Bureau (USCB). United States Current Population Survey, June 2010. Washington, D.C., United States of America: United States Census Bureau (USCB).

17. United States Census Bureau (USCB). United States Current Population Survey, July 2010. Washington, D.C., United States of America: United States Census Bureau (USCB).

18. United States Census Bureau (USCB). United States Current Population Survey, August 2010. Washington, D.C., United States of America: United States Census Bureau (USCB).

19. United States Census Bureau (USCB). United States Current Population Survey, September 2010. Washington, D.C., United States of America: United States Census Bureau (USCB).

20. United States Census Bureau (USCB). United States Current Population Survey, October 2010. Washington, D.C., United States of America: United States Census Bureau (USCB).

21. United States Census Bureau (USCB). United States Current Population Survey, November 2010. Washington, D.C., United States of America: United States Census Bureau (USCB).

22. United States Census Bureau (USCB). United States Current Population Survey, December 2010. Washington, D.C., United States of America: United States Census Bureau (USCB).

23. United States Census Bureau (USCB). United States Current Population Survey, January 2011. Washington, D.C., United States of America: United States Census Bureau (USCB).

24. United States Census Bureau (USCB). United States Current Population Survey, February 2011. Washington, D.C., United States of America: United States Census Bureau (USCB).

25. United States Census Bureau (USCB). United States Current Population Survey, March 2011. Washington, D.C., United States of America: United States Census Bureau (USCB).

26. United States Census Bureau (USCB). United States Current Population Survey, April 2011. Washington, D.C., United States of America: United States Census Bureau (USCB).

27. United States Census Bureau (USCB). United States Current Population Survey, May 2011. Washington, D.C., United States of America: United States Census Bureau (USCB).

28. United States Census Bureau (USCB). United States Current Population Survey, June 2011. Washington, D.C., United States of America: United States Census Bureau (USCB).

29. United States Census Bureau (USCB). United States Current Population Survey, July 2011. Washington, D.C., United States of America: United States Census Bureau (USCB).

30. United States Census Bureau (USCB). United States Current Population Survey, August 2011. Washington, D.C., United States of America: United States Census Bureau (USCB).

31. United States Census Bureau (USCB). United States Current Population Survey, September 2011. Washington, D.C., United States of America: United States Census Bureau (USCB).

32. United States Census Bureau (USCB). United States Current Population Survey, October 2011. Washington, D.C., United States of America: United States Census Bureau (USCB).

33. United States Census Bureau (USCB). United States Current Population Survey, November 2011. Washington, D.C., United States of America: United States Census Bureau (USCB).

34. United States Census Bureau (USCB). United States Current Population Survey, December 2011. Washington, D.C., United States of America: United States Census Bureau (USCB).

35. United States Census Bureau (USCB). United States Current Population Survey, January 2012. Washington, D.C., United States of America: United States Census Bureau (USCB).

36. United States Census Bureau (USCB). United States Current Population Survey, February 2012. Washington, D.C., United States of America: United States Census Bureau (USCB).

37. United States Census Bureau (USCB). United States Current Population Survey, March 2012. Washington, D.C., United States of America: United States Census Bureau (USCB).

38. United States Census Bureau (USCB). United States Current Population Survey, April 2012. Washington, D.C., United States of America: United States Census Bureau (USCB).

39. United States Census Bureau (USCB). United States Current Population Survey, May 2012. Washington, D.C., United States of America: United States Census Bureau (USCB).

40. United States Census Bureau (USCB). United States Current Population Survey, June 2012. Washington, D.C., United States of America: United States Census Bureau (USCB).

41. United States Census Bureau (USCB). United States Current Population Survey, July 2012. Washington, D.C., United States of America: United States Census Bureau (USCB).

42. United States Census Bureau (USCB). United States Current Population Survey, August 2012. Washington, D.C., United States of America: United States Census Bureau (USCB).

43. United States Census Bureau (USCB). United States Current Population Survey, September 2012. Washington, D.C., United States of America: United States Census Bureau (USCB).

44. United States Census Bureau (USCB). United States Current Population Survey, October 2012. Washington, D.C., United States of America: United States Census Bureau (USCB).

45. United States Census Bureau (USCB). United States Current Population Survey, November 2012. Washington, D.C., United States of America: United States Census Bureau (USCB).

46. United States Census Bureau (USCB). United States Current Population Survey, December 2012. Washington, D.C., United States of America: United States Census Bureau (USCB).

47. United States Census Bureau (USCB). United States Current Population Survey, January 2013. Washington, D.C., United States of America: United States Census Bureau (USCB).

48. United States Census Bureau (USCB). United States Current Population Survey, February 2013. Washington, D.C., United States of America: United States Census Bureau (USCB).

49. United States Census Bureau (USCB). United States Current Population Survey, March 2013. Washington, D.C., United States of America: United States Census Bureau (USCB).

50. United States Census Bureau (USCB). United States Current Population Survey, April 2013. Washington, D.C., United States of America: United States Census Bureau (USCB).

51. United States Census Bureau (USCB). United States Current Population Survey, May 2013. Washington, D.C., United States of America: United States Census Bureau (USCB).

52. United States Census Bureau (USCB). United States Current Population Survey, June 2013. Washington, D.C., United States of America: United States Census Bureau (USCB).

53. United States Census Bureau (USCB). United States Current Population Survey, July 2013. Washington, D.C., United States of America: United States Census Bureau (USCB).

54. United States Census Bureau (USCB). United States Current Population Survey, August 2013. Washington, D.C., United States of America: United States Census Bureau (USCB).

55. United States Census Bureau (USCB). United States Current Population Survey, September 2013. Washington, D.C., United States of America: United States Census Bureau (USCB).

56. United States Census Bureau (USCB). United States Current Population Survey, October 2013. Washington, D.C., United States of America: United States Census Bureau (USCB).

57. United States Census Bureau (USCB). United States Current Population Survey, November 2013. Washington, D.C., United States of America: United States Census Bureau (USCB).

58. United States Census Bureau (USCB). United States Current Population Survey, December 2013. Washington, D.C., United States of America: United States Census Bureau (USCB).

59. United States Census Bureau (USCB). United States Current Population Survey, January 2014. Washington, D.C., United States of America: United States Census Bureau (USCB).

60. United States Census Bureau (USCB). United States Current Population Survey, February 2014. Washington, D.C., United States of America: United States Census Bureau (USCB).

61. United States Census Bureau (USCB). United States Current Population Survey, March 2014. Washington, D.C., United States of America: United States Census Bureau (USCB).

62. United States Census Bureau (USCB). United States Current Population Survey, April 2014. Washington, D.C., United States of America: United States Census Bureau (USCB).

63. United States Census Bureau (USCB). United States Current Population Survey, May 2014. Washington, D.C., United States of America: United States Census Bureau (USCB).

64. United States Census Bureau (USCB). United States Current Population Survey, June 2014. Washington, D.C., United States of America: United States Census Bureau (USCB).

65. United States Census Bureau (USCB). United States Current Population Survey, July 2014. Washington, D.C., United States of America: United States Census Bureau (USCB).

66. United States Census Bureau (USCB). United States Current Population Survey, August 2014. Washington, D.C., United States of America: United States Census Bureau (USCB).

67. United States Census Bureau (USCB). United States Current Population Survey, September 2014. Washington, D.C., United States of America: United States Census Bureau (USCB).

68. United States Census Bureau (USCB). United States Current Population Survey, October 2014. Washington, D.C., United States of America: United States Census Bureau (USCB).

69. United States Census Bureau (USCB). United States Current Population Survey, November 2014. Washington, D.C., United States of America: United States Census Bureau (USCB).

70. United States Census Bureau (USCB). United States Current Population Survey, December 2014. Washington, D.C., United States of America: United States Census Bureau (USCB).

71. United States Census Bureau (USCB). United States Current Population Survey, January 2015. Washington, D.C., United States of America: United States Census Bureau (USCB).

72. United States Census Bureau (USCB). United States Current Population Survey, February 2015. Washington, D.C., United States of America: United States Census Bureau (USCB).

73. United States Census Bureau (USCB). United States Current Population Survey, March 2015. Washington, D.C., United States of America: United States Census Bureau (USCB).

74. United States Census Bureau (USCB). United States Current Population Survey, April 2015. Washington, D.C., United States of America: United States Census Bureau (USCB).

75. United States Census Bureau (USCB). United States Current Population Survey, May 2015. Washington, D.C., United States of America: United States Census Bureau (USCB).

76. United States Census Bureau (USCB). United States Current Population Survey, June 2015. Washington, D.C., United States of America: United States Census Bureau (USCB).

77. United States Census Bureau (USCB). United States Current Population Survey, July 2015. Washington, D.C., United States of America: United States Census Bureau (USCB).

78. United States Census Bureau (USCB). United States Current Population Survey, August 2015. Washington, D.C., United States of America: United States Census Bureau (USCB).

79. United States Census Bureau (USCB). United States Current Population Survey, September 2015. Washington, D.C., United States of America: United States Census Bureau (USCB).

80. United States Census Bureau (USCB). United States Current Population Survey, October 2015. Washington, D.C., United States of America: United States Census Bureau (USCB).

81. United States Census Bureau (USCB). United States Current Population Survey, November 2015. Washington, D.C., United States of America: United States Census Bureau (USCB).

82. United States Census Bureau (USCB). United States Current Population Survey, December 2015. Washington, D.C., United States of America: United States Census Bureau (USCB).

83. United States Census Bureau (USCB). United States Current Population Survey, January 2016. Washington, D.C., United States of America: United States Census Bureau (USCB).

84. United States Census Bureau (USCB). United States Current Population Survey, February 2016. Washington, D.C., United States of America: United States Census Bureau (USCB).

85. United States Census Bureau (USCB). United States Current Population Survey, March 2016. Washington, D.C., United States of America: United States Census Bureau (USCB).

86. United States Census Bureau (USCB). United States Current Population Survey, April 2016. Washington, D.C., United States of America: United States Census Bureau (USCB).

87. United States Census Bureau (USCB). United States Current Population Survey, May 2016. Washington, D.C., United States of America: United States Census Bureau (USCB).

88. United States Census Bureau (USCB). United States Current Population Survey, July 2016. Washington, D.C., United States of America: United States Census Bureau (USCB).

89. United States Census Bureau (USCB). United States Current Population Survey, August 2016. Washington, D.C., United States of America: United States Census Bureau (USCB).

90. United States Census Bureau (USCB). United States Current Population Survey, September 2016. Washington, D.C., United States of America: United States Census Bureau (USCB).

91. United States Census Bureau (USCB). United States Current Population Survey, October 2016. Washington, D.C., United States of America: United States Census Bureau (USCB).

92. United States Census Bureau (USCB). United States Current Population Survey, November 2016. Washington, D.C., United States of America: United States Census Bureau (USCB).

93. United States Census Bureau (USCB). United States Current Population Survey, December 2016. Washington, D.C., United States of America: United States Census Bureau (USCB).

94. United States Census Bureau (USCB). United States Current Population Survey, January 2017. Washington, D.C., United States of America: United States Census Bureau (USCB).

95. United States Census Bureau (USCB). United States Current Population Survey, February 2017. Washington, D.C., United States of America: United States Census Bureau (USCB).

96. United States Census Bureau (USCB). United States Current Population Survey, March 2017. Washington, D.C., United States of America: United States Census Bureau (USCB).

97. United States Census Bureau (USCB). United States Current Population Survey, April 2017. Washington, D.C., United States of America: United States Census Bureau (USCB).

98. United States Census Bureau (USCB). United States Current Population Survey, May 2017. Washington, D.C., United States of America: United States Census Bureau (USCB).

99. United States Census Bureau (USCB). United States Current Population Survey, June 2017. Washington, D.C., United States of America: United States Census Bureau (USCB).

100. United States Census Bureau (USCB). United States Current Population Survey, July 2017. Washington, D.C., United States of America: United States Census Bureau (USCB).

101. United States Census Bureau (USCB). United States Current Population Survey, August 2017. Washington, D.C., United States of America: United States Census Bureau (USCB).

102. United States Census Bureau (USCB). United States Current Population Survey, September 2017. Washington, D.C., United States of America: United States Census Bureau (USCB).

103. United States Census Bureau (USCB). United States Current Population Survey, October 2017. Washington, D.C., United States of America: United States Census Bureau (USCB).

104. United States Census Bureau (USCB). United States Current Population Survey, November 2017. Washington, D.C., United States of America: United States Census Bureau (USCB).

105. United States Census Bureau (USCB). United States Current Population Survey, December 2017. Washington, D.C., United States of America: United States Census Bureau (USCB).

106. United States Census Bureau (USCB). United States Current Population Survey, January 2018. Washington, D.C., United States of America: United States Census Bureau (USCB).

107. United States Census Bureau (USCB). United States Current Population Survey, February 2018. Washington, D.C., United States of America: United States Census Bureau (USCB).

108. United States Census Bureau (USCB). United States Current Population Survey, March 2018. Washington, D.C., United States of America: United States Census Bureau (USCB).

109. United States Census Bureau (USCB). United States Current Population Survey, April 2018. Washington, D.C., United States of America: United States Census Bureau (USCB).

110. United States Census Bureau (USCB). United States Current Population Survey, May 2018. Washington, D.C., United States of America: United States Census Bureau (USCB).

111. United States Census Bureau (USCB). United States Current Population Survey, June 2018. Washington, D.C., United States of America: United States Census Bureau (USCB).

112. United States Census Bureau (USCB). United States Current Population Survey, July 2018. Washington, D.C., United States of America: United States Census Bureau (USCB).

113. United States Census Bureau (USCB). United States Current Population Survey, August 2018. Washington, D.C., United States of America: United States Census Bureau (USCB).

114. United States Census Bureau (USCB). United States Current Population Survey, September 2018. Washington, D.C., United States of America: United States Census Bureau (USCB).

115. United States Census Bureau (USCB). United States Current Population Survey, October 2018. Washington, D.C., United States of America: United States Census Bureau (USCB).

116. United States Census Bureau (USCB). United States Current Population Survey, November 2018. Washington, D.C., United States of America: United States Census Bureau (USCB).

117. United States Census Bureau (USCB). United States Current Population Survey, December 2018. Washington, D.C., United States of America: United States Census Bureau (USCB).

118. United States Census Bureau (USCB). United States Current Population Survey, January 2019. Washington, D.C., United States of America: United States Census Bureau (USCB).

119. United States Census Bureau (USCB). United States Current Population Survey, February 2019. Washington, D.C., United States of America: United States Census Bureau (USCB).

120. United States Census Bureau (USCB). United States Current Population Survey, March 2019. Washington, D.C., United States of America: United States Census Bureau (USCB).

121. United States Census Bureau (USCB). United States Current Population Survey, April 2019. Washington, D.C., United States of America: United States Census Bureau (USCB).

122. United States Census Bureau (USCB). United States Current Population Survey, May 2019. Washington, D.C., United States of America: United States Census Bureau (USCB).

123. United States Census Bureau (USCB). United States Current Population Survey, June 2019. Washington, D.C., United States of America: United States Census Bureau (USCB).

124. United States Census Bureau (USCB). United States Current Population Survey, July 2019. Washington, D.C., United States of America: United States Census Bureau (USCB).

125. United States Census Bureau (USCB). United States Current Population Survey, August 2019. Washington, D.C., United States of America: United States Census Bureau (USCB).

126. United States Census Bureau (USCB). United States Current Population Survey, September 2019. Washington, D.C., United States of America: United States Census Bureau (USCB).

127. United States Census Bureau (USCB). United States Current Population Survey, October 2019. Washington, D.C., United States of America: United States Census Bureau (USCB).

128. United States Census Bureau (USCB). United States Current Population Survey, November 2019. Washington, D.C., United States of America: United States Census Bureau (USCB).

129. United States Census Bureau (USCB). United States Current Population Survey, December 2019. Washington, D.C., United States of America: United States Census Bureau (USCB).

130. Bureau of Labor Statistics, U.S. Department of Labor, Occupational Employment Statistics, [10/20/2021] [www.bls.gov/oes/].

131. Bureau of Labor Statistics, U.S. Department of Labor, Occupational Employment Statistics, [10/20/2021] [www.bls.gov/oes/].

132. Bureau of Labor Statistics, U.S. Department of Labor, Occupational Employment Statistics, [10/20/2021] [www.bls.gov/oes/].

133. Bureau of Labor Statistics, U.S. Department of Labor, Occupational Employment Statistics, [10/20/2021] [www.bls.gov/oes/].

134. Bureau of Labor Statistics, U.S. Department of Labor, Occupational Employment Statistics, [10/20/2021] [www.bls.gov/oes/].

135. Bureau of Labor Statistics, U.S. Department of Labor, Occupational Employment Statistics, [10/20/2021] [www.bls.gov/oes/].

136. Bureau of Labor Statistics, U.S. Department of Labor, Occupational Employment Statistics, [10/20/2021] [www.bls.gov/oes/].

137. Bureau of Labor Statistics, U.S. Department of Labor, Occupational Employment Statistics, [10/20/2021] [www.bls.gov/oes/].

138. Bureau of Labor Statistics, U.S. Department of Labor, Occupational Employment Statistics, [10/20/2021] [www.bls.gov/oes/].

139. Bureau of Labor Statistics, U.S. Department of Labor, Occupational Employment Statistics, [10/20/2021] [www.bls.gov/oes/].

140. Inter-university Consortium for Political and Social Research (ICPSR), Johns Hopkins Bloomberg School of Public Health, Westat. National Health and Aging Trends Study 2011. National Health and Aging Trends Study (NHATS).

141. Inter-university Consortium for Political and Social Research (ICPSR), Johns Hopkins Bloomberg School of Public Health, Westat. National Health and Aging Trends Study 2012. National Health and Aging Trends Study (NHATS).

142. Inter-university Consortium for Political and Social Research (ICPSR), Johns Hopkins Bloomberg School of Public Health, Westat. National Health and Aging Trends Study 2013. National Health and Aging Trends Study (NHATS).

143. Inter-university Consortium for Political and Social Research (ICPSR), Johns Hopkins Bloomberg School of Public Health, Westat. National Health and Aging Trends Study 2014. National Health and Aging Trends Study (NHATS).

144. Inter-university Consortium for Political and Social Research (ICPSR), Johns Hopkins Bloomberg School of Public Health, Westat. National Health and Aging Trends Study 2015. National Health and Aging Trends Study (NHATS).

145. Inter-university Consortium for Political and Social Research (ICPSR), Johns Hopkins Bloomberg School of Public Health, Westat. National Health and Aging Trends Study 2016. National Health and Aging Trends Study (NHATS).

146. Inter-university Consortium for Political and Social Research (ICPSR), Johns Hopkins Bloomberg School of Public Health, Westat. National Health and Aging Trends Study 2017. National Health and Aging Trends Study (NHATS).

147. Inter-university Consortium for Political and Social Research (ICPSR), Johns Hopkins Bloomberg School of Public Health, Westat. National Health and Aging Trends Study 2018. National Health and Aging Trends Study (NHATS).

148. Inter-university Consortium for Political and Social Research (ICPSR), Johns Hopkins Bloomberg School of Public Health, Westat. National Health and Aging Trends Study 2019. National Health and Aging Trends Study (NHATS).

149. United States Census Bureau (USCB). United States Historical Income Tables: Median Household Income by State 1984-2021. Washington, D.C., United States of America: United States Census Bureau (USCB), 2022.

150. Eli Lilly and Company. United States Longitudinal Cohort Study of Resource Use and Cost of Mild Cognitive Impairment and Mild Dementia Due to Alzheimer's Disease 2016-2021.

151. Genworth Financial. United States Cost of Care Survey 2012. Richmond, United States of America: Genworth Financial.

152. Genworth Financial. United States Cost of Care Survey 2015. Richmond, United States of America: Genworth Financial.

153. Genworth Financial. United States Cost of Care Survey 2018. Richmond, United States of America: Genworth Financial.

154. Ong KL, Stafford LK, McLaughlin SA, et al. Global, regional, and national burden of diabetes from 1990 to 2021, with projections of prevalence to 2050: a systematic analysis for the Global Burden of Disease Study 2021. *The Lancet*. 2023;402(10397):203-234. doi:10.1016/S0140-6736(23)01301-6

155. Friedman J, York H, Graetz N, et al. Measuring and forecasting progress towards the education-related SDG targets. *Nature*. 2020;580(7805):636-639. doi:10.1038/s41586-020-2198-8
